# Ultrafast lasers for attosecond science

**DOI:** 10.1038/s41377-025-02121-4

**Published:** 2026-01-02

**Authors:** Xijie Hu, Ka Fai Mak, Jinwei Zhang, Zhiyi Wei, Ferenc Krausz

**Affiliations:** 1 School of Optical and Electronic Information and Wuhan National Laboratory for Optoelectronics, Huazhong University of Science and Technology, 430074 Wuhan, China; 2 Max Planck Institute of Quantum Optics, Hans-Kopfermann-Str. 1, 85748 Garching, Germany; 3 Institute of Physics, Chinese Academy of Sciences, Beijing, 100190 China

**Keywords:** Optical techniques, Optical physics

## Abstract

The first measurement of attosecond pulses in 2001 unleashed a new wave of exploration in the microcosmic world. The pulse width has since shrunk from an initial 650 to 43 as, and the flux, photon energy, and repetition rates have progressively been raised. The performance of attosecond pulses hinges upon the driving lasers, whose rapid development underlaid many advancements of attosecond technology. Yet the expansion of new applications in attosecond science demands driving lasers with ever better performance. Beginning with the fundamental principles of attosecond pulse generation and applications, this article reviews the evolution and trend of the driving lasers in terms of pulse energy, pulse width, wavelength, and repetition rate.

## Introduction

The attosecond pulse^1–3^, currently the shortest event attainable and controllable by mankind, unlocks previously inaccessible capability to probe the microcosm. The ability to directly investigate ultrafast processes at their native attosecond-scale^4–7^ heralds an exciting new chapter in the understanding of matter. Significant progress in both science and technology has been ensured, with extensive prospects for applications in transient absorption spectroscopy^8–14^, nonlinear attosecond experiments^15,16^, ultrafast electron dynamics^17–20^, condensed matter physics^21–23^, chemistry^24–26^, with exciting potentials for other multidisciplinary applications in biology and energy science.

Currently, attosecond pulses are primarily generated through high-order harmonic generation (HHG), which has been successfully demonstrated in various media, including gases, solids, and plasmas. Although the underlying mechanisms differ among these media, the generation process fundamentally arises from the nonlinear electronic response induced by intense laser fields. Specifically, it can be understood as the ionization of an electron by the laser’s electric field, whose oscillation accelerates the freed electron first away, and then back towards the ion. The recombination of the electron and ion releases the gained kinetic energy, in addition to the binding energy, in the form of an attosecond XUV light pulse. The properties of such pulses are critically influenced by the driving laser’s parameters, namely pulse duration, pulse energy, wavelength, and repetition rate. Shorter driver pulse durations, corresponding to fewer electric-field oscillation cycles, reduce the number of acceleration and recombination events, facilitating the generation of isolated attosecond pulses. Higher single-pulse energies enhance the ionizing field strength and increase the efficiency of attosecond generation. Longer driving wavelengths, with longer path for electron acceleration, significantly increase the HHG cutoff energy but substantially reduce the harmonic conversion efficiency. Higher repetition rates can improve the signal-to-noise ratio and data acquisition efficiency, but are often constrained by limited pulse energy for the driving pulse, and thereby the attosecond pulse.

The diverse attosecond applications each require distinct properties of attosecond light^27^. These, in turn, demand driving femtosecond laser sources with varied characteristics as mentioned above. For example, isolated attosecond pulses (IAPs) that are highly beneficial for ultrafast dynamics^28^ and electron microscopy^29^ require driving pulses lasting a mere few optical cycles. High-energy attosecond pulses, crucial for pump-probe spectroscopy^30^ and multi-photon ionization^31^ research, depend on plasma-based high-harmonic generation (HHG) in the highly ionized regime, which calls for high-energy, multi-mJ-level driving lasers. For generating attosecond pulses in the X-ray water window—valuable for applications such as coherent imaging^32^ and time-resolved X-ray absorption spectroscopy^33^—drivers with longer wavelengths in the mid-infrared are necessary to maximize the photon energy and raise the central frequency of the attosecond pulses^34^. Applications such as coincidence measurement^35^ and photoemission spectroscopy^36^ benefit from attosecond pulses with high repetition rates (>10 kHz) but lower pulse energies^37^ to achieve high signal-to-noise ratios and avoid spatial charge effects.

**Fig. 1:**
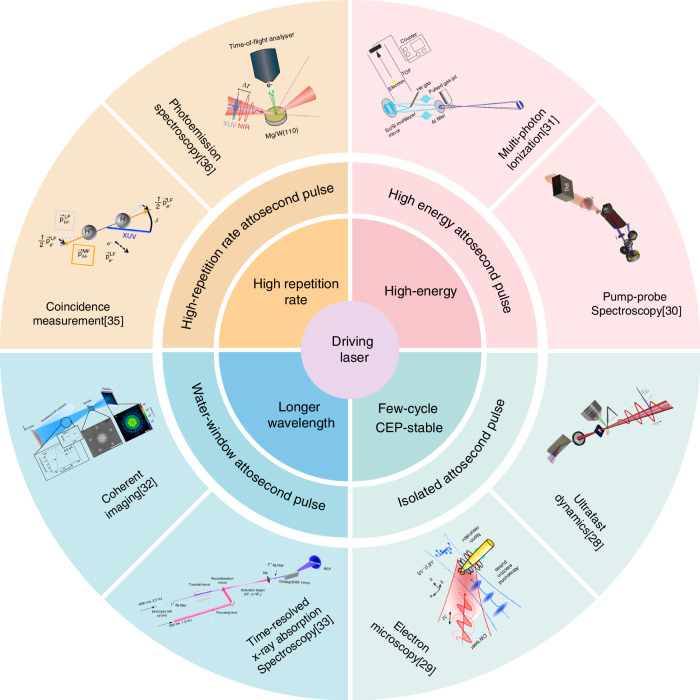
**Relationship between attosecond pulse applications and driving laser parameters**. Adapted from ref.^28–31,35,36^, with permission from Springer Nature. Copyright © 2004–2023. Reproduced from ref.^32^ under a Creative Commons Attribution 4.0 International License (CC BY 4.0); Adapted from ref.^33^, with permission from AAAS. Copyright © 2017

The close relationship between the driving laser, the attosecond pulses and their relevant applications is shown in Fig. 1. The applications' ever more stringent demands require further improvement in attosecond pulse parameters, which in turn motivates new developments in the corresponding driving lasers.

### Laser parameters for attosecond pulse generation

Unlike femtosecond or picosecond pulses, attosecond pulses cannot be generated using conventional laser cavities. Instead, they are generated using methods such as HHG^38,39^, Thomson scattering^40–42^, Raman scattering^43^, coherent synthesis of femtosecond lasers^44–46^, free electron lasers (FELs)^47^, diffraction-limited storage ring (DLSR)^48,49^, and soliton compression dynamics^50^. Among the various techniques, HHG remains the most established and widely adopted method for attosecond pulse generation. Based on different physical mechanisms, HHG can be classified into three types: gas-phase HHG, solid- and liquid-phase HHG, and plasma-surface HHG.

#### In gas medium

Currently, the method most widely used is HHG in gases^51^. In this scheme, ultra-intense femtosecond pulses interact with gas atoms and molecules, driving extreme nonlinear processes to generate high harmonics and the corresponding attosecond pulses. In 1993, Paul Corkum proposed the three-step model^52^ to describe the process of high harmonics generation at the single-atom level. Similar models were also proposed by Kulander^53^ and Schafer^54^ around the same time. The three-step model predicts how the parameters of a continuous wave laser field— its wavelength and intensity—will affect the generated high harmonics given the temporal periodicity of the electric field. For a continuous wave laser, every half-cycle of the laser field is identical. In each half-cycle, the emission of high harmonics occurs through three steps: (1) Strong-field ionization: in order to overcome the coulomb potential that an electron experiences in the ground state, the laser intensity should be sufficient (>10^13^ W∙cm^−2^) to partially ionize the atoms or molecules. This results in the liberation of an electron wavepacket into the continuum. (2) Drift in the laser field: the electron wavepacket, freed through ionization, undergoes acceleration due to the laser's electric field and accumulates kinetic energy. The longer the laser's wavelength, the longer the time that the electron is accelerated in the continuum and the higher the kinetic energy the electron can accumulate at a given intensity. The wavepacket will also be broader in space, reducing the probability of the following step. (3) High harmonic generation: during the drift, the electron may be pulled back by the laser field, recombining with the host ion and subsequently releasing its kinetic energy plus the ionization potential as a photon: high harmonic radiation. Thus, the photon energy of the harmonics is higher for longer driving wavelengths, but the broader wavepacket also leads to lower efficiency.

This process depends on not just the laser’s wavelength and intensity, but on the temporal evolution of its electric field. In a typical femtosecond laser pulse, the duration of the envelope encompasses several oscillations of the carrier wave. Thus, it is insufficient to consider only a single half-cycle for HHG; the duration and the carrier-envelope phase (CEP) of the pulse also play important roles. Each field half-cycle above the threshold of ionization generates a high harmonic pulse, forming an attosecond pulse train (APT). If the CEP—the relative phase between the envelope of the laser pulse and the carrier electric field—varies between each driving pulse, the resulting high harmonics will have differing spectra and widths due to the varying electric field distributions^55^. To obtain the same APT for each driving pulse, the CEP needs to be stabilized.

As the driving laser pulse duration shortens, there will be fewer optical cycles and fewer attosecond pulses in the APT. The spectral width of each harmonic will correspondingly increase. When the pulse duration reaches a single cycle, the high harmonics spectrum merges into a supercontinuum^56^ and forms an isolated attosecond pulse in the time domain^1^. However, achieving such a single-cycle driving laser proves challenging in experiments.

Employing gating techniques can reduce the requirements for the driving laser's duration, enabling the production of IAPs using driving lasers with one to two cycles or even multiple cycles. Take amplitude gating as an example. If the CEP of a few-cycle laser pulse is close to zero^57^ and there exists one dominating half-cycle, one can isolate the spectral portion in the cutoff region to form an isolated attosecond pulse. Common gating techniques for attosecond pulses encompass amplitude gating^58–61^, ionization gating^62^, polarization gating^63–65^, double optical gating (DOG)^66^, attosecond lighthouse^67^ and interferometric polarization gating^68^.

#### In liquid and solid targets

High-order harmonics and attosecond pulses can also be generated from solids, which involves two main principles^69^. One is radiation resulting from nonlinearities in the intraband current^70–72^, including through Bloch oscillations^73^. The other is emission from interband polarization^74–76^, which resembles the three-step model of HHG from gas. In solid materials, intense laser fields prompt electrons to transition from the valence band to the conduction band, creating holes in the valence band. Subsequently, electrons and holes drift at the corresponding group velocities under the influence of the strong laser field. Eventually, due to lattice scattering and the intense laser field, electrons return from the conduction band to the valence band, combining with holes and emitting high-frequency radiation. A more comprehensive description and analysis can be found in the review by Shambhu Ghimire et al.^69^ and Eleftherios Goulielmakis et al.^77^. Unlike high harmonics from gases, where the cutoff energy is proportional to the square of the wavelength^52,78^, the cutoff energy of solid high harmonics scales linearly with the field amplitude^69,79^.

Although high harmonics in liquid have been experimentally realized^80–82^, the underlying principle of liquid HHG remains a topic of debate^83^. Chang-Long Xia et al. proposed a quantum theory that successfully explains most of the known properties of liquid harmonics and suggests that the liquid high-harmonic cutoff energy is independent of the driving laser wavelength^83^. Tran Trung Luu et al. experimentally observed that the cutoff photon energy of liquid high harmonics is linearly related to the peak electric field strength^82^.

#### In plasma

When the intensity of the driving laser is increased to 10^16^ W∙cm^-2^ ^84^, another effective method for generating high harmonics and attosecond pulses is via ultra-intense laser ablation of solids^85^ and solid surface plasmas^86,87^ with full ionization. These involve two distinct mechanisms^86,88^. The first relies on the relativistic Doppler upshift of the laser light reflected off an oscillating plasma surface that acts as a relativistic oscillating mirror (ROM)^89,90^. The other mechanism arises from currents excited by fast electrons in the density ramp of the plasma-vacuum interface, known as coherent wake emission (CWE)^91^. The conversion efficiency of HHG from solid-state plasmas can reach up to 10^-4^, surpassing that of gas by more than one order of magnitude. The method of generating attosecond laser pulses by driving laser ablation of plasmonic nanostructures has also been theoretically demonstrated^92,93^, but it has not yet been achieved experimentally. This remains one of the directions for future exploration in attosecond science.

### The requirements for next-generation attosecond sources

The development of attosecond pulses has been making great strides. Currently, the shortest attosecond pulse width has been reduced to 43 as^61^. The maximum pulse energy of an IAP reaches up to 1.3 μJ, with a corresponding intensity at gigawatt-level^15^. The maximum pulse energy of APTs reaches up to 7.15 μJ, providing an intensity of 11 gigawatts^94^. The highest photon energy extends to the water window region, reaching up to 1.6 keV^95^. Additionally, the maximum repetition rate has reached 1 MHz^96^.

**Fig. 2:**
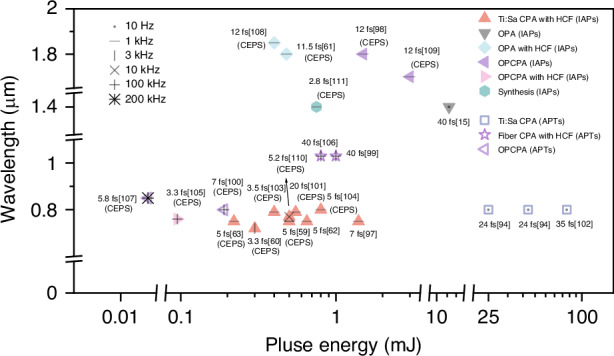
**Typical driving lasers for generating attosecond pulses**. From refs. ^15,59–63,94,103,365,389,451,453,475,479,480,496–502^

In essence, the nonlinear interaction between the driving lasers and various target matter underlies the generation of high harmonics and attosecond laser pulses. Regardless of the target type, the driving laser plays a crucial role in determining the characteristics of the generated attosecond pulses. A single driving laser is unlikely to simultaneously meet the diverse requirements of various attosecond applications, and selecting driving lasers with matching characteristics is essential. Based on the demands of frontier technologies and applications such as pump-probe spectroscopy^30^, electron microscopy^29^, coherent imaging^32^, attosecond lasers are expected to develop towards higher-energy attosecond pulses, IAPs, attosecond pulses in the X-ray water window, and high-repetition rate attosecond pulses. This, in turn, calls for driving lasers with higher pulse energies, shorter pulse widths, longer wavelengths, and increased repetition rates. Figure 2 summarizes the performance of various driving lasers currently capable of generating attosecond pulses. This paper will discuss the historical development and trends of driving lasers from these four aspects.

## Research progress

### High-energy driving lasers for high-energy attosecond pulses

Since the first realization of the laser in 1960^97^, its performance has seen colossal improvement. The pulse duration directly obtainable from a laser oscillator has shortened by a remarkable 12 orders of magnitude, from microseconds when free running, to nanoseconds with Q-switching, and to picoseconds and femtoseconds with mode-locking. The obtainable pulse energy has also reached formidable levels, from micro-joules emitted directly from an oscillator to multiple joules after further amplification. These laid the foundation for high-field physics, the generation of high-harmonics and made possible the realization of attosecond pulses^98,99^.

The HHG process demands substantial power densities from the driving laser to induce highly nonlinear ionizing effects in the gas target, with requirements exceeding 10^13^ W∙cm^−2 ^^100^. Even when employing solid targets, the power density threshold stands at 10^11^ W∙cm^−2^. And when utilizing laser-produced plasmas, it escalates to 10^16^ W∙cm^−2^ ^84^. Furthermore, the laser's focusing area—constrained by the laser's wavelength—cannot be infinitely reduced. Reaching the required power densities necessitates having driving pulses with high peak power, i.e., driving pulses with higher energies and shorter durations.

Adding to the challenges, the conversion efficiency of HHG is very low, ranging typically from 10^−8^ to 10^−4^ depending on the laser parameters and the desired energy. The low efficiency results in generally low attosecond pulse energies, with only a rare few examples reaching the nano-joule level^101^. This restricts their utility in various fields, including pump-probe spectroscopy^30^, coherent diffractive imaging^102^, single-order ultrafast imaging and multi-photon ionization^31,103^. Consequently, the boosting of photon flux is a major part of the ongoing attosecond research effort. This compels the corresponding advancement in driving laser technology toward elevated pulse energies. Currently, the primary methods for attaining high driver pulse energies involve amplification and coherent pulse combination. These will be introduced below.

#### Amplification

Achieving a high peak power to drive attosecond generation begins with amplifying femto- and pico-second pulses. Commonly employed amplification techniques include chirped pulse amplification (CPA) and optical parametric amplification (OPA), together with their combination and derivatives: optical parametric chirped pulse amplification (OPCPA), double-chirped optical parametric amplification (DC-OPA), frequency domain optical parametric amplification (FOPA) and quasi-parametric chirped-pulse amplification (QPCPA). The research community involved in these developments is large and constantly growing. Here, we will summarize only some of the main concepts and results. For a more extensive and in-depth review, we refer the reader to the recent article by Chang et al^104^.

##### Chirped pulse amplification (CPA)

The concept of CPA was initially introduced in 1985 by Mourou and Strickland at the University of Rochester. They obtained 2 ps pulses with 1 mJ of pulse energy by transposing amplification techniques from the radar domain^105^. This groundbreaking work earned them the Nobel Prize in Physics in 2018. Building upon CPA, femtosecond laser systems have achieved peak powers exceeding 10 PW, covering a wide range of intensities from 10^14^ to 10^25^ W∙cm^−2^^106^.

CPA systems play a pivotal role in boosting the energy of ultrashort pulses. Before CPA's emergence, high-energy pulses were commonly generated via direct amplification. However, the excessively high peak intensity in the laser amplifier often surpassed the damage threshold of the amplifying gain medium. The only means to prevent component damage was by expanding the focus spot and enlarging the diameter of the gain medium. As a result, the practical dimensions of the gain medium and optical components impose limitations on the development of laser pulse energy.

A typical CPA system consists of an oscillator, stretcher, amplifier, and compressor. The stretcher initially introduces dispersion to the ultra-short femtosecond or picosecond pulses produced by the oscillator. This dispersion increases the pulse duration to hundreds of picoseconds or even nanoseconds in the time domain, significantly reducing the peak power while maintaining energy density per unit area. Subsequently, the stretched pulse undergoes amplification and is compressed back through a compressor, compensating for the introduced dispersion and restoring the pulse width to the femtosecond or picosecond range. The CPA system not only overcomes the challenges of damaging optical components or crystals in the beamline during amplification but also mitigates adverse nonlinear effects such as gain saturation. This capability enables the rapid development of high-pulse-energy laser systems. CPA technology, particularly when employing Ti:sapphire, stands as the primary method for generating high-energy laser pulses due to its high efficiency and stability. To further enhance the energy output, one approach is to scale up the size of the laser crystals^107,108^, while another method involves employing multiple amplification stages^109,110^. Presently, pulse energies generated using CPA technology can already reach hundreds of joules^108,110^. However, the average power for Ti:sapphire-based systems is constrained by the large quantum defect of the gain medium^111^. Furthermore, due to the non-uniform gain of the amplifying media across the pulse spectrum and gain narrowing, high-power CPA systems encounter challenges in generating few-cycle pulses. The average output power can be raised to kilowatt-level by using rare-earth-doped gain media such as Yb:YAG. They exhibit a lower quantum defect and can be shaped into various geometries that are advantageous for heat dissipation, such as fiber, rod, slab, and thin disk. In particular, thin-disk-based CPAs^112–114^ combine efficient thermal management with high peak-power handling and good spatial beam quality, enabling them to sustain kW-level average power output while delivering ultrashort pulses with energies reaching 720 mJ^113^. Nevertheless, their narrow gain bandwidth can only support pulse durations at or longer than hundreds of femtosecond. To reach the high-power, few-cycle regime, post-compression techniques, introduced in the section “Nonlinear post-compression technique”, are to be combined with CPA systems.

##### Optical parametric amplification (OPA)

OPA dates back to the 1960s^115^—soon after the first laser emerged—and is primarily utilized for creating frequency-tunable sources. The pump laser and the signal laser to be amplified are concurrently focused into a suitable nonlinear crystal, which facilitates the transfer of energy from the high-frequency, high-energy pump laser to the low-frequency, low-energy signal laser. From a photon perspective, OPA involves the absorption of the high-frequency photon (pump photon) and the excitation of the nonlinear crystal to a virtual energy level, followed by its decay stimulated by the low-frequency signal photon. The signal photon is thereby replicated (the signal is amplified), together with the generation of an additional idler photon at the frequency difference between the pump and the signal^116^. The signal and idler photons may or may not be of the same frequency, representing the degenerate and non-degenerate cases, respectively. Since the process does not require real absorption in the nonlinear crystal, there is minimal thermal load on the crystal.

The realization of the potential broadband gain inherent in the parametric process, coupled with the emergence of boronic acid crystals^117^ featuring high nonlinear coefficients and excellent dispersion characteristics, fueled the rapid evolution of OPA schemes aimed at producing high-power, few-cycle laser pulses. The highest pulse energy attainable with OPA technology at present is demonstrated in 2015 by Nicolas Thiré and colleagues, producing 10-mJ pulses lasting 30 fs (five cycles) at a wavelength of 1.8 μm and a repetition rate of 100 Hz, driven by a Ti-Sapphire laser system^118^. Despite OPA technology lagging behind CPA technology in terms of pulse energy amplification, its wavelength tunability renders it remarkably versatile, finding applications across both visible^119,120^ and infrared spectra^121^, with particularly notable performance in the infrared range^122–124^. In contrast to CPA, where parasitic pre-pulses are often generated and requires additional suppression techniques^125–130^, OPA inherently provides improved pulse contrast by amplifying only within the time window defined by the pump pulse duration, usually in the femtosecond range. In addition, OPA's high parametric gain and broad wavelength tuning range make it an excellent tool for generating high-power mid-infrared pulses—effective for driving HHG that targets significantly higher cutoff frequencies.

Furthermore, OPAs produce pulses that can be compressed to nearly a single optical cycle. Thus, mainstream HHG driving lasers have gradually transitioned from Ti-sapphire lasers to mid-infrared optical parametric amplifiers^39^. However, optical damage and the size of nonlinear crystals in OPA systems restrict the energy of the pump light and, consequently, the pulse energy achievable from OPA. The pulse energy is typically limited to the microjoule scale, and it has been challenging to exceed 10 millijoules^118,124^. To mitigate damage to nonlinear crystals induced by high-energy pump lasers, subsequent researchers have proposed various schemes, including OPCPA, FOPA, and DC-OPA.

##### Optical parametric chirped pulse amplification (OPCPA)

In 1992, the OPCPA technique was introduced^131^, combining OPA and CPA techniques. OPCPA inherits the benefits of both OPA and CPA, including broad gain bandwidth, low thermal loading, low b-integral, high amplification gain, high signal-to-noise ratio, wavelength flexibility, and the capability to increase energy without damaging nonlinear crystals. In the OPCPA scheme, the broadband seed pulse is initially chirped and temporally broadened, and synchronized with a pump pulse of similar pulse durations. Subsequently, the pulse undergoes parametric amplification in a nonlinear crystal and, finally, compression to yield ultrashort pulses. OPCPA can achieve high-power amplification on the order of multiple joules. In contrast to CPA, it facilitates high-power amplification of few-cycle pulses. Depending on the phase-matching configuration between the signal and the pump pulse, OPCPA technology is categorized into collinear and non-collinear, the latter being known as non-collinear optical parametric chirped pulse amplification (NOPCPA).

Presently, laser pulses stemming from OPCPA systems can approach 50 J^132^ and conversion efficiencies can exceed 40%^133^. OPCPA demonstrates the capability to generate pulses with ultra-high intensities exceeding 10^23^ W∙cm^−2^. The wavelengths of these pulses can span from the visible to the infrared region^134–137^. Moreover, this technique has successfully led to the generation of IAPs^138^. In addition, the millijoule-scale output of the OPCPA unit can be used as a seed for additional CPA units, generating pulses with hundred-joule-scale output^139,140^ and PW-level peak power with relatively high signal-to-noise ratios (the strength of the main pulse compared to background noise) and contrast (the strength of the main pulse compared to any parasitic pre- or post-pulses).

While the performance of OPCPA has improved tremendously, a trade-off exists between achieving a high conversion efficiency and attaining an ultra-wide amplified bandwidth, which is contingent upon the chirp of the seed pulse^141,142^. Additionally, noise stemming from spontaneous parametric generation, or “superfluorescence”, emerges as a primary limiting factor in signal energy scaling, particularly in high-gain OPCPA stages^143,144^. This may surpass signal amplification, leading to a considerable decrease in the energy and stability of the amplified signal^141^. However, in low-gain stages with sufficiently strong seed injection—such as the final stages of multi-stage OPCPA systems—the influence of superfluorescence is considerably reduced and is not a dominant constraint on energy scaling.

##### Dual-chirped optical parametric amplification (DC-OPA)

**Fig. 3:**
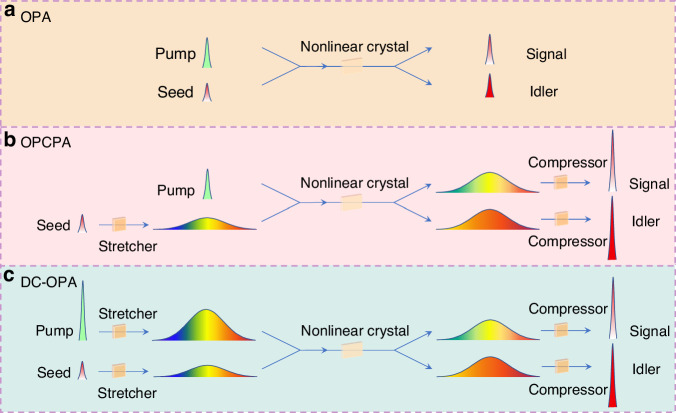
Schemes of three parametric amplification configurations. **a** OPA; **b** OPCPA; **c** DC-OPA. Redrawn based on ref.^145^

In 2011, Zhang et al. introduced a theoretical concept known as DC-OPA, aiming to generate high-power infrared pulses with few-cycle pulse durations^145^. Unlike OPCPA, where only the seed pulse undergoes stretching, in the DC-OPA system, both the pump and seed pulses have broad spectral bandwidth and are chirped. Figure 3 illustrates a schematic comparison of OPA, OPCPA, and DC-OPA.

Compared to the conventional OPCPA configuration, the DC-OPA system provides greater flexibility in broadband phase-matching and thereby larger parametric gain bandwidth due to the variable chirp of the pump pulses. Using such a technique, 44 fs infrared pulses with a total energy exceeding 210 mJ has been achieved, at a repetition rate of 10 Hz^146^. Furthermore, coupled with Ti:sapphire lasers with even higher pulse energies at tens or even hundreds of joules^110^, DC-OPA has the potential to scale the energy of infrared pulses to 10 J while preserving a wide spectral bandwidth.

**Fig. 4:**
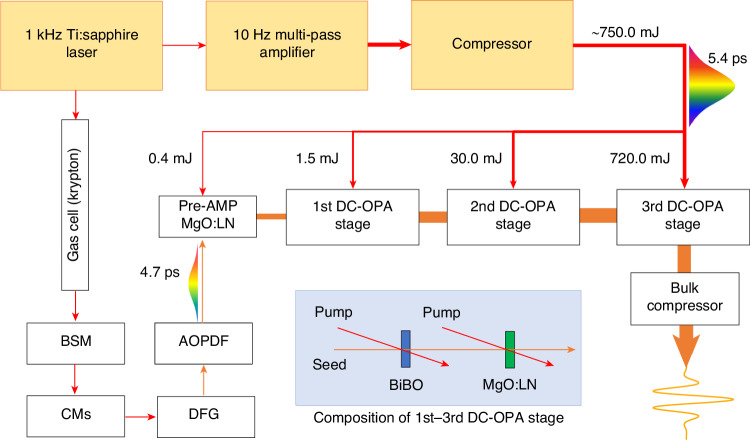
**Example setup of a high-energy, single-cycle MIR laser source based on the advanced DC-OPA scheme**^150^. Reproduced from ref.^150^, licensed under CC BY 4.0

Additionally, DC-OPA excels in generating few-cycle pulses. To achieve a broader spectrum supporting a signal pulse with few-cycle transform-limited durations, two variants of DC-OPA are used. One method involves employing a pump laser with a broad spectrum^135,147,148^. The other focuses on using crystals with a broader phase-matching bandwidth^146^. BiB_3_O_6_ (BiBO)^149^ and YCa_4_O (BO_3_)_3_ (YCOB)^146^ crystals are commonly used nonlinear crystals known for their wide phase-matching bandwidths. They facilitate an already impressive output pulse duration of sub-two cycles. To overcome the gain bandwidth limitation inherent in a single nonlinear crystal, Lu Xu and Eiji J. Takahashi further optimized the approach by demonstrating an advanced DC-OPA scheme utilizing both BiBO and MgO-doped lithium niobate crystals, as depicted in Fig. 4. This configuration produced CEP-stable mid-infrared laser pulses spanning a bandwidth exceeding one octave (1.4–3.1 μm), corresponding to 1.05 cycles (8.58 fs) at 2.44 μm^150^. These pulses achieved an output pulse energy of up to 53 mJ with a peak power of 6 TW, representing the pinnacle of achievement in optical parametric amplification for producing single-cycle mid-infrared laser pulses.

##### Frequency domain optical parametric amplification (FOPA)

**Fig. 5:**
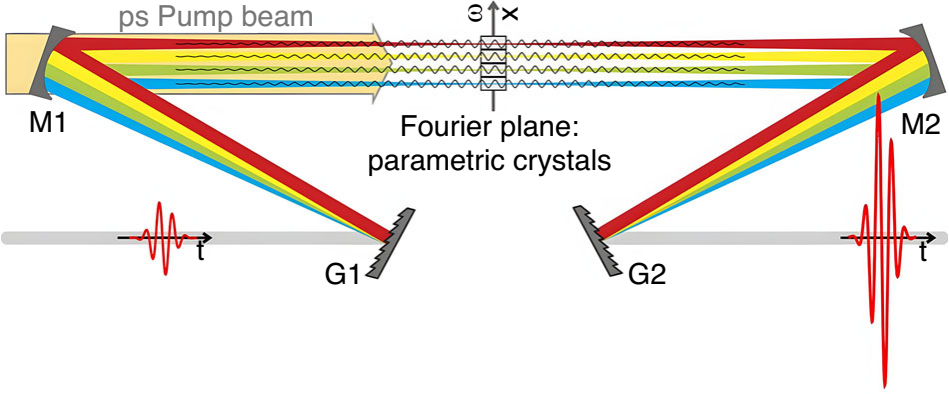
**Concept of FOPA**^151^. Reproduced from ref.^151^, licensed under CC BY 4.0

In contrast to stretching the pulse temporally to overcome intensity limitation, an alternative approach is to disperse the frequency components spatially. In the FOPA scheme proposed by Bruno E. Schmidt et al., the input pulse undergoes spectral dispersion in a symmetric 4-*f* system^151^ (Fig. 5). The various frequencies in the Fourier plane (FP) are incident on multiple individually tunable nonlinear crystals for “slice-by-slice” amplification. Subsequently, a second Fourier transform occurs in the same mirror-grating arrangement as the pulse exits the FOPA, restoring, in the time domain, a pulse of the same duration as the input. Two-cycle CEP-stable pulses with a pulse energy of 1.43 mJ and a central wavelength of 1.8 μm has been achieved based on this technique^151^.

This scheme overcomes limitations stemming from phase-matching bandwidth and damage thresholds of an individual crystal. By increasing the number of crystals, FOPA can amplify more of the seed spectrum or energy, enhancing both pulse energy and amplification bandwidth simultaneously. It also avoids the uncompensated spectral phase introduced by thermal-lensing effects^151,152^, and suppresses hyperfluorescent background in the output beam^152^. In contrast to the conventional CPA technique^153^, the signal in an FOPA does not exhibit spatial chirp at the gratings. As a result, the FOPA output is free from coherent noise typically caused by the finite aperture^154^ and surface roughness^155,156^ of gratings used in traditional stretchers and compressors. Instead, the temporal coherent noise originates from spatial imperfections during amplification, which may be more conducive to achieving high coherent-contrast pulses^157^.

**Fig. 6:**
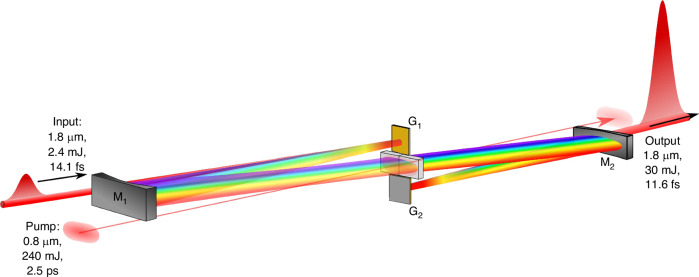
**FOPA with a non-collinear geometry**^158^. Reprinted with permission from ref.^158^ © Optica Publishing Group

Presently, FOPA-based lasers have demonstrated the capability to attain pulse energies of 30 mJ and peak powers of 2.5 TW by employing two barium borate (BBO) crystals and a 240 mJ titanium sapphire pump laser (Fig. 6)^158^. Moreover, the FOPA system shows promise for achieving higher pulse energies and peak powers, potentially reaching the order of 10 TW with the utilization of joule-sized pump pulses.

However, employing the FOPA system is not without drawbacks. It demands meticulous alignment of the optical path length in each crystal, with intricacy increasing with the number of crystals used. Moreover, diffraction from the edges of the crystals can introduce pre-pulses^159^. The spectral bandwidth and efficiency of FOPA also depend heavily on the grating, and it is challenging to fabricate a highly efficient grating across a wide spectral range^151^.

##### Quasi-parametric chirped-pulse amplification (QPCPA)

A key bottleneck in OPA and its derived schemes is the reversibility of the three-wave mixing processes, where both amplification and back-conversion share the same energy conservation and phase-matching conditions. As the intensities of the signal and idler waves increase, the reverse process becomes inevitable, leading to a “backflow” of pump energy, which severely limits both efficiency and stability^141,160^.

To address the limitation, the quasi-parametric chirped-pulse amplification (QPCPA) scheme has been proposed^161^. This architecture preserves the broadband and high-gain advantages of OPCPA while fundamentally suppressing back-conversion by selectively attenuating the idler, thereby breaking energy reversibility. A typical implementation involves doping the nonlinear crystal with rare-earth ions (e.g., Sm³⁺) to enable absorption of the idler. Using this approach, experiments have demonstrated 85% pump depletion, 56% signal conversion efficiency, and output pulses with energies of 65 mJ and durations around 100 fs. Under optimal conditions, pump depletion can approach 96%, and signal conversion efficiency can reach up to 63%^162^.

The absorption of the idler disrupts the energy flow among the three interacting waves, relaxing the phase-matching criteria. This broadens the amplification bandwidth and enhances gain uniformity, enabling QPCPA to amplify broadband few-cycle pulses^163^ with a theoretical pulse duration compressible to ~10 fs^162^. Moreover, QPCPA’s strong tolerance to phase mismatch makes it resistant to thermal dephasing effects and suitable for high-average-power operation. When combined with a high-energy pump source, the system is expected to achieve joule-level energy output and petawatt-level peak power^162^.

However, QPCPA remains at the validation stage and faces numerous challenges. Currently, high-quality and scalable absorptive nonlinear crystals are still scarce; aside from Sm³⁺-doped YCa_4_O(BO_3_)_3_ (Sm:YCOB) crystals^162^, no mature material systems exist. Sm³⁺-doped La_3_Ga_5.5_Nb_0.5_O_14_ (Sm:LGN) crystals have been employed in simulations for mid-infrared QPCPA^164^, but have not yet been experimentally verified. Notably, non-absorptive idler dissipation mechanisms based on upconversion processes such as second-harmonic generation (SHG) or sum-frequency generation (SFG) have recently been proposed as alternative approaches to rare-earth-doped crystals^165^. This strategy facilitates the expansion of signal wavelength ranges and crystal types while avoiding additional thermal load, thus supporting high-average-power QPCPA systems. Yet, this approach remains theoretical.

Nevertheless, by introducing an energy irreversibility mechanism, QPCPA achieves an excellent balance between efficiency, bandwidth, and thermal stability. This provides a novel approach to generating few-cycle, high-power ultrafast laser systems and is one of the key developmental directions for next-generation high-energy ultrafast light sources.

A comparison of experimental parameters for OPA, OPCPA, FOPA, and DC-OPA techniques is presented in Table 1^146^. As QPCPA remains in the experimental validation stage and lacks comprehensive, systematic data, it has been excluded from this table.

**Table 1:** Characteristics of OPA, OPCPA, FOPA, and DC-OPA. Adapted from ref.^146^, licensed under CC BY 4.0

	OPA^146^	OPCPA^132–134,146,493^	FOPA^146,151,158^	DC-OPA^146^
Pump source	fs Ti:sapphire CPA	ps laser	fs Ti:sapphire CPA	fs Ti:sapphire CPA
Pump duration	TL	TL	Chirped	Chirped
Pump spectrum	Broad	Narrow	Broad	Broad
Maximum pump energy	0.1-J-level	200-J-level	10-J-level	100-J-level
Seed duration	TL	Chirped	TL	Chirped
Seed spectrum	Broad	Broad	Broad	Broad
Energy scaling	Difficult	Straightforward	Possible	Straightforward
Output pulses	Signal and idler	Signal and idler	Signal	Signal and idler
conversion efficiency	30–40%	10–41%	14%	30–40%
Highest reported output energy	10 mJ	45 J	30 mJ	210 mJ
few-cycle pulse generation	Yes	Yes	Yes	Yes
wavelength tunability	Excellent	Good	Not reported	Excellent
How a broad output spectrum is efficiently generated	Thin crystal with broad phasematched bandwidth.	Thin crystal with broad phasematched bandwidth. Pump duration on the 1 ps level.	Phase-matched bandwidth of the crystal. Number of crystals in the Fourier plane.	Phase-matched bandwidth of the crystal. Chirp management of pump and seed pulses.
Compressor	Not needed	Needed	Not needed	Most often but not always needed

#### Coherent beam combining

Coherently adding multiple lower-power beams together is an alternative way to scale up the power beyond what is achievable from an individual high-power laser limited by thermal and nonlinear problems^166^. As early as 2005, T. Y. Fan et al. proposed the method to enhance the output power of continuous lasers^167^. The idea was subsequently applied to pulsed lasers. Currently, there are two routes to obtaining high-energy pulses by adding pulses coherently: coherent beam combination (CBC), which divides the pulse in space, and divided-pulse amplification (DPA), which divides the pulse in time.

##### Coherent beam combination (CBC)

**Fig. 7:**
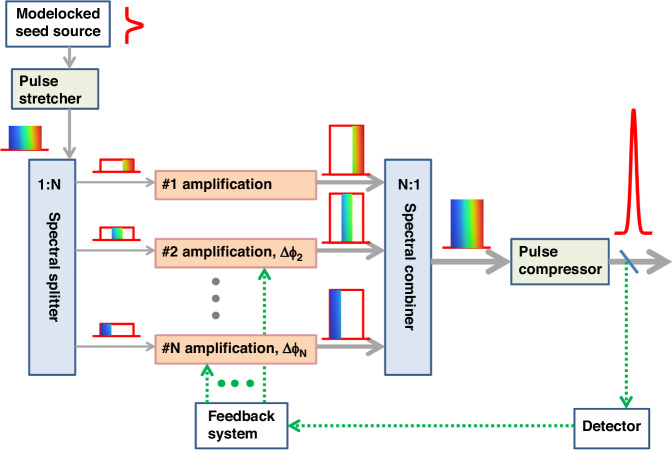
**Schematic of a coherent beam combination setup including an active control of the optical phase**^168^. Adapted with permission from ref.^168^. © Optica Publishing Group

CBC involves dividing the pulses from the same ultrashort pulse laser oscillator into multiple beams, which are amplified in separate channels and later coherently recombined, before finally being temporally compressed—the schematic is shown in Fig. 7^168^. Coherent beam combination is mostly used in fiber amplification because the good and stable beam quality of a fiber amplifier allows for efficient coherent overlapping of the beam profile. By using amplifiers with different emission bands, the technology offers a way to overcome gain-narrowing limitations in a single fiber amplifier^168^.

The first experimental demonstration coherent pulse combining sums the output of two-fiber-based CPA systems^169^. The initial iteration of this system showed a high efficiency of 97%, with subsequent advances having led to astounding improvements in pulse energies and power. Through strategies such as augmenting amplification channels and employing active pulse shaping techniques, the output average power of CBC-based lasers has surged beyond kilowatts^170^, while pulse energy has reached the millijoules scale^171,172^. Peak power now exceeds 20 GW^173^, and pulse width has been significantly reduced to the order of a hundred femtoseconds^174^. However. with this method, the more amplifier channels and the more complex the structure, the more difficult it is to control the system, and its synthesis efficiency and beam quality are limited by the thermal lensing effect of the optics.

##### Divided-pulse amplification (DPA)

**Fig. 8:**
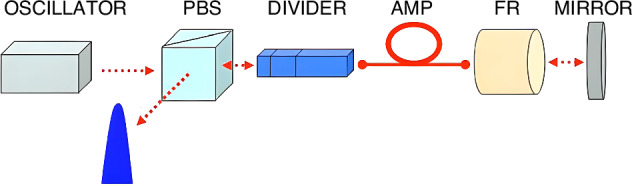
**Schematic of a DPA setup**^175^. Reprinted with permission from ref.^175^ © Optica Publishing Group

DPA shares the same concept of pulse splitting with CBC, but does so in the temporal domain. Using a birefringent pulse splitter, DPA divides the input pulse into multiple temporally separated sub-pulses and thereby reduces nonlinear effects during amplification. The sub-pulses are automatically recombined into a single high-energy pulse after propagation through the system. A schematic of the DPA experiment is shown in Fig. 8. Since its first demonstration^175^, the technique has been adapted to different laser systems^176–180^, and also combined with the CBC technique^181^.

The DPA technology can be categorized as passive DPA and active DPA. In passive DPA, a single interferometer or element is used for both pulse splitting and recombination. The different pulses pass through the same optical path and are automatically recombined into a single pulse. The pulse energy output of passive DPA systems can reach microjoules^176,178,181^ with a maximum reported output of 10 mJ^182^. However, the sub-pulses can have small energy differences which, in combination with the saturation of the amplifier, means they have different amplitudes and will accumulate different nonlinear phases, restricting their efficient recombination. Thus, passive DPA proves ineffective at higher energies^183–185^. The performance of DPA can be improved by actively shaping the input sub-pulse’s amplitude and phase, a technique known as active DPA^186^. Output pulse energies based on this technique can exceed a hundred millijoule^187^.

To further boost the overall amplification, both DPA and CBC can be employed concurrently. In CBC, the beam undergoes spatial splitting into *N* beams, which are individually amplified in spatially separated amplifiers. DPA temporally splits pulses into *M* replicas. Integrating both spatial and temporal multiplexing approaches can yield a scalable *N·M* multidimensional amplification scheme^188^. An example is a main amplifier stage comprising up to eight spatially separated amplifier channels, with each amplifying four temporally divided-pulse replicas^189^. However, DPA faces limitations in efficiently supporting the numerous sub-pulses due to restricted access to individual sub-pulses' phase and amplitude—crucial for compensating amplifier saturation and nonlinearity^190^. To address this, an innovative approach named electro-optically controlled divided-pulse amplification (EDPA) was proposed^191^. This method allows for precise control over the amplitude and phase of each sub-pulse within a burst, realized in a compact fiber-integrated frontend. The EDPA was subsequently implemented into an existing high-power ultrafast fiber laser system based on CBC. This implementation involved spatio-temporal pulse addition of 8-pulse bursts from up to 12 parallel amplifiers, achieving a combined power of 674 W, an energy of 23 mJ in the main pulse, at a combining efficiency of 71%^190^.

DPA and CBC for ultrashort pulses are mainly used in fiber-optic or fiber-like solid-state amplifiers and face similar issues in managing pulse splitting, amplification and recombination^187^. Their main challenge is in maintaining efficiency during recombination, with any mismatch in pulse parameters leading to losses and efficiency degradation^192^.

### Few-cycle CEP-stable driving lasers—for IAP generation

IAPs, with their exceptionally broad spectra and ultrashort durations, are powerful tools for studying ultrafast dynamics in matter^4,28^, including via time-resolved x-ray absorption spectroscopy (TR-XAS)^33,193–195^. The generation of IAPs relies on high-power pulses lasting only a few optical cycles in duration, which in turn requires a sufficiently broad optical spectrum with a relatively constant spectral phase. In practice, it is challenging for a laser medium to generate pulses that simultaneously possess high power and broad spectral bandwidth. This is partially due to the gain-narrowing effect during amplification and the strong thermal effects in common broadband gain medium such as Ti:sapphire crystals. Therefore, a typical approach involves first generating a pulse with high power but a relatively narrow spectral width and subsequently broadening its spectrum and compensating its spectral phase to reach the desired few-cycle pulse duration. This strategy is often known as nonlinear post-compression.

#### Nonlinear post-compression technique

The spectral broadening in post-compression is brought about by propagating an intense light pulse through a nonlinear medium, resulting in phase distortion and the emergence of new spectral components. Nonlinear post-compression techniques widely employed for generating few-cycle pulses include hollow-core fibers (HCF), multiple-plate supercontinuum compression (MPSC), and multipass cells (MPC). Currently, these techniques can support post-compression of pulses with energies up to 200 mJ^112^. In addition, these techniques can be further combined with downstream waveform synthesizers to obtain sub-fs pulses^44,196^. Table 2 presents the state-of-the-art parameters based on these techniques without additional waveform synthesis. For joule-level pulse energies, a thin film is also used as nonlinear medium^197–202^. Nevertheless, since the driving lasers for attosecond pulse generation typically do not require such high energy levels, we will not discuss this technique further.

**Table 2:** Research status of HCF, MPSC, and MPC for generating few-cycle pulses

Post-compression Technique	HCF	MPSC	MPC
Maximum input pulse energy (mJ)	70^210^	1.07^236^	200^112^
Maximum output pulse energy (mJ)	40^210^	0.68^230^	150^112^
Maximum input average power (W)	580^213^	80^238^	1045^261^
Maximum output average power (W)	318^213^	35^238^	1004^261^
Shortest post-compression pulse Width (fs)	3.2^218^/0.9^223^^a^	2.6^235^	6.9^494^
Fewest post-compression pulse Periods	1.5^218,219^	<1(0.94)^236^	2^494^
Maximum compression factor	40^225^	53^236^	125^263^
Highest post-compression efficiency (%)	71.1^495^	85.0^239^	96.4^255^

^a^0.9 fs is achieved with soliton self-compression^223^, whereas 3.2 fs is obtained using conventional HCF-compression^218^

Table 2 reveals that, among the HCF, MPSC and MPC technologies, MPC currently supports the highest average laser power and pulse energy, enabling kilowatt-level and 150-millijoule pulse compression, respectively. Following this, HCF technology facilitates sub-kilowatt and sub-100 millijoule pulse compression, while MPSC technology enables pulse compression approaching 100 W and at the millijoule scale. Notably, nonlinear pulse compression using HCF, specifically with soliton self-compression, has achieved the shortest durations reported to date, reaching 0.9 fs and entering the sub-femtosecond regime. Meanwhile, MPSC has also demonstrated pulse durations as short as 2.6 fs at a central wavelength of 800 nm, enabling the generation of sub-cycle optical pulses. MPC technology demonstrates the highest compression ratio, exceeding 125, and achieves the highest broadening compression efficiency of up to 96.4%.

Below, we will introduce the development and pros and cons of these three post-compression techniques. A more in-depth discussion on these and other techniques can also be found in reference^203^.

##### Hollow-capillary fiber (HCF)

**Fig. 9:**
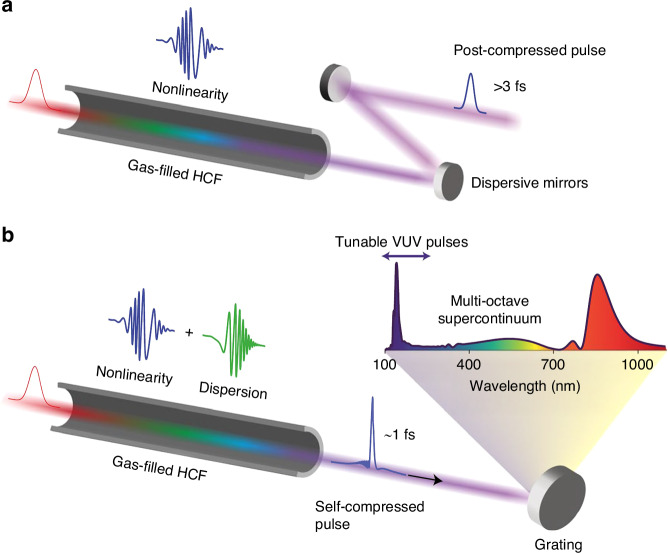
**Schematic of pulse compression using HCF technology**. **a** Conventional post-compression with a gas-filled HCF; **b** Soliton self-compression dynamics in a gas-filled HCF^50^. Reproduced from ref.^50^, with permission from Springer Nature. Copyright © 2019 Springer Nature

In 1996, M. Nisoli proposed compression using HCF. By focusing the laser light into a gas-filled hollow-core fiber to broaden the spectrum and compression using chirped mirrors, ultra-short pulses of 240 μJ and 10 fs were obtained^204^. The use of gases instead of solids as dielectric media significantly raises the damage threshold and laser intensity that can be sustained. Specifically, inert gases are employed to ensure pure electronic response (i.e., no Raman contribution) and the highest possible ionization threshold. Common gas choices include Ar^205^, Ne^206,207^, Kr^208,209^, and He^210^, with He exhibiting the highest ionization threshold, albeit having the lowest nonlinearity. The wave-guiding property of hollow-core fiber, in turn, offers a long interaction length and enables self-phase modulation to occur uniformly across the transvers beam profile^211^. Propagation lengths can span multiple meters when stretched flexible HCFs^212^ are used. The typical structure is illustrated in Fig. 9a. This method, known for its broad spectrum and excellent output beam quality, has been extensively used and developed since its inception and remains the predominant compression method in the few-cycle field.

Currently, through HCF-compression, the maximum pulse energy achievable is 40 mJ^210^, and the maximum average output power is 318 W^213^. The primary limitation in further scaling the pulse energy lies in gas ionization within the HCF, which is partially mitigated by a pressure gradient^214^ and the use of circularly polarized pulses^215,216^. As the available driver laser pulse energy continues to increase, fibers with larger cores (>1 mm) have also been used^217^, leading to a compressed peak power of 1.3 TW^210^. Apart from raising the output pulse energy, research efforts have also been devoted to obtaining shorter pulse durations. Presently, the shortest attainable pulse width via traditional HCF compression is 3.2 fs^218^, and a minimum pulse period of 1.5 cycles^218,219^. By pre-shaping the pulse before it enters the HCF, single-cycle pulses down to 2.2 fs has also been demonstrated^220^.

Recently, novel schemes of pulse compression based on HCF but without the need of subsequent phase compensation have also been proposed and experimentally demonstrated. In 2019, J. Travers et al. demonstrated for the first time soliton dynamics in an HCF, as illustrated in Fig. 9b^50^, which represents an important shift in paradigm in HCF compression technique^221^. It involves balancing the total dispersion of the gas-filled waveguide with the nonlinear self-phase modulation to invoke soliton self-compression of the propagating pulse, resulting in sub-cycle (2 fs) pulses with 27 GW of peak power directly exiting the fiber^222^. Recently, the technique has been demonstrated to provide sub-femtosecond pulses, setting new records for the shortest pulses achievable with current nonlinear post-compression techniques^223^. The self-compression dynamic has also been scaled to TW-level^224^. Additionally, these fibers can directly generate ultraviolet radiation and support the formation of multi-octave-spanning supercontinua. Another technique that directly generates ultrashort pulses at the end of a gas-filled HCF is by taking advantage of the nonlinear mixing of spatial modes during propagation. This converts some of the input energy in the near-IR pulse (175 fs, 1 mJ, centered at 1035 nm) into a pulse in the visible region (4.6 fs, 20 µJ, at 600 nm)^225^.

One potential weakness of the HCF technique lies in its average power handling. When the power exceeds several hundreds of Watts, the HCF needs to be cooled to reduce the thermal effects on the straightness of the HCF^213,226^.

##### Multiple-plate supercontinuum compression (MPSC)

**Fig. 10:**
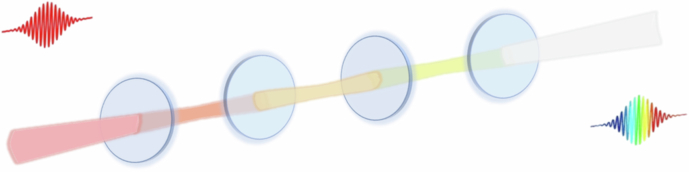
**Illustration showing the multiple plates arrangement consisting of four thin fused silica plates aligned at Brewster’s angle and located at the waist of the incident beam**. Redrawn based on ref.^228^

The concept of spectrally broadening femtosecond laser pulses by integrating multiple fused silica plates and apertures was first proposed by Voronin et al. in 2013^227^ and experimentally realized by Lu et al. in 2014^228^. Employing four 100-μm-thick UV-fused silica plates placed at Brewster angles (see Fig. 10), a 140 μJ, 25-fs pulse produced more than one octave of spectral broadening, spanning 450 to 980 nm. Pushing the concept further, a white light continuum of 235 μJ was generated from a 25 fs pulse through six 20-μm-thin plates. Subsequent temporal compression to 9 fs by chirped mirrors facilitated the successful generation of several orders of high harmonic pulses in argon^229^. Building upon these advancements, a near-transform-limited duration of 2.8 fs was generated with a 160 µJ pulse, close to a single optical cycle^230^.

The fundamental purpose of employing multiple solid plates to achieve few-cycle laser pulses is to circumvent catastrophic beam collapse due to self-focusing and ensure the effect of self-phase modulation is spread evenly across the transverse mode of the beam. When a pulse's peak power exceeds the critical self-focusing power of the nonlinear medium it is propagating through, the self-focusing effect overpowers diffraction and the spot size diminishes progressively. With a long enough distance, it can result in the collapse of the beam and damage to the solid material^231^. The utilization of multiple thin plates interspersed with air allows strong self-phase modulation to occur at power beyond the critical self-focusing power. Although the beam still experiences self-focusing, it exits the thin medium before beam collapse occurs, and the resulting focus is in the air between plates, where the nonlinearity is much lower and no permanent damage is induced. The beam then diverges due to diffraction and enters the next thin medium, which imparts additional self-phase modulation and refocuses the beam again. The periodic focusing and divergence maintain the spot size within a stable range through the multiple plates^232,233^. As each plate contributes to the total B-integral, a sequence of such thin plates can still facilitate significant broadening and generate a supercontinuum. When the nonlinear effects within the crystal plates reach a dynamic balance with the linear dispersion and diffraction between the plates, a quasi-stationary structure resembling soliton-like propagation can emerge. This structure helps maintain the spatial mode quality of the input pulse^234^. However, in MPSC systems, such soliton-like behavior can only be sustained under specific conditions. As a result, MPSC systems may still experience beam quality degradation, often manifested as spatial distortions such as ring-like features and inhomogeneous beam profiles^230,235^.

At present, pulses obtained using the MPSC post-compression technique can already be compressed to a single cycle^235–237^. Compared to HCF systems, the MPSC method offers numerous advantages, including high efficiency, stability, compact structure, simplified alignment, and flexibility. However, compared to using HCFs and MPCs, the MPSC method has the drawback of being less energy scalable. The achievable output pulse energy is so far limited to sub-mJ level^235,238,239^, with the maximum pulse energy reported being 0.68 mJ^230^. This limitation stems from its dependence on condensed materials with high nonlinearities^203,240^. Furthermore, the multiplate approach is also limited by a practical number of passes. The requirement for using higher nonlinear phase accumulation per pass also leads to imperfect spectral homogeneity and a small amount of spatial chirp in the output beam^203,240^.

##### Multipass cell (MPC)

Multipass spectral broadening in bulk materials was first investigated with regenerative amplifiers^241–243^. Good spatial beam quality was also predicted for pulses that underwent multiple passes through a nonlinear medium for self-phase modulation^244^. The first demonstration of spectral broadening using Herriott-type MPC^245^, which has become the standard cell type nowadays, was proposed in 2015^246^ and later demonstrated as part of an amplifier system^247^.

**Fig. 11:**
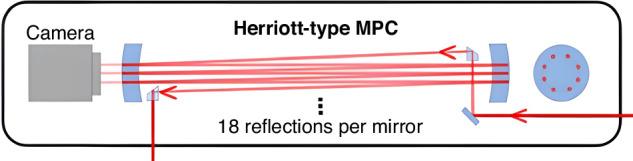
**The structure of the MPC**^248^. Adapted with permission from ref.^248^ © Optica Publishing Group

The first work focusing on employing the MPC for nonlinear post-compression was reported by Schulte et al.^248^ in 2016. A Herriott-type MPC, comprising a convex surface coated with a highly reflective (HR) coating and a concave surface coated with an anti-reflection (AR) coating (see Fig. 11) was used. Additionally, the HR specular coating is dispersive to compensate for substrate material dispersion. Schulte achieved pulse durations of 170 fs at an output power of 375 W with a transmission rate of 70.75% using the MPC. This was accomplished by spectrally broadening input pulses from a 10 MHz, 850 fs, 530 W Yb:YAG-Innoslab laser system from 1.6 to over 13.5 nm bandwidth.

In the MPC scheme, two (or more) mirrors are arranged such that they host transversal eigenmodes, analogous to passive laser cavities. Off-axis propagation on spherical mirrors, e.g., in Herriott cells, enables multipass operation with virtually lossless in- and out-coupling. The MPC maintains beam parameters and functions as a “discrete” waveguide^203^. Laser pulses are repeatedly propagated through a thin solid or extended gaseous nonlinear medium, generating a small B-integral during each pass. Maintaining a low single-pass B-integral is crucial for preserving constant beam parameters and aligning with the cavity^249–251^. This process enables pulses with peak powers even surpassing the critical self-focusing threshold by orders of magnitude to undergo spectral broadening with minimal beam quality degradation^252^. As the pulses undergo multiple (tens of) passes, the nonlinear phase accumulates, enabling significant spectral broadening.

Nonlinear pulse compression using MPC offers superior power scalability and optical efficiency compared to other techniques. Its limitations stem primarily from the damage threshold and the size of the MPC, as well as coating damage and thermal distortion caused by laser light absorption in the nonlinear medium.

Depending on the requirements, both solid and gas can be used as the nonlinear medium. Fused silica is initially utilized for relatively low-energy lasers, resulting in pulse broadening capabilities typically limited to the micro-Joule range^248,250,251,253^. However, a recent study has demonstrated that, through optimization of system design and experimental conditions, the use of fused silica can broaden pulses with up to 2 mJ of pulse energy^254^. The use of gas as the nonlinear medium facilitates pulse broadening routinely on the multi-millijoule scale^249,255–259^. The removal of discrete nonlinear elements for gas-filled MPCs delocalizes the nonlinear response and strongly mitigates the impact of varying self-phase modulation across the transverse beam profile. Consequently, the gas-filled approach enables approximately five times higher accumulated nonlinear phase per pass than with discrete nonlinear elements, thus promoting stronger spectral broadening^255^.

The first-order helical Laguerre–Gaussian mode can further enhance the energy throughput of nonlinear spectral broadening in gas-filled multipass cells, pushing the obtainable output pulse energy to 107.8 mJ^260^. Based on the fundamental laser mode, Yanik Pfaff et al. demonstrated nonlinear spectral broadening exceeding 150 mJ pulse energy using a Herriott-type multipass cell^112^. This marks the highest pulse energy achieved not only for using MPCs, but among all nonlinear post-compression techniques. Besides, the MPC technique stands as the first and only post-compression method enabling sub-50-fs compression at kW-level average power. Christian Grebing et al. demonstrated the reliable generation of 1 mJ, 31 fs pulses with an average power of 1004 W through post-compression of 200 fs, 1045 W pulses from a coherently combined Yb:fiber laser system. This was achieved in an argon-filled Herriott-type multipass cell with an overall compression efficiency of 96%^261^. This output represents the highest average power of sub-100-fs pulses ever demonstrated and showcases the MPC's capability to deliver kilowatt pulses.

In addition, the MPC demonstrates the capability to achieve remarkably high compression ratios, reducing picosecond pulses to less than 50 fs using only one MPC stage^258^. By cascading multiple MPCs, even greater compression factor can be attained^262^, compressing picosecond pulses to less than 10 fs at a compression factor of up to 125^263^. The current duration record based on MPCs technology is 5.8 fs^264^ and corresponds to around two optical cycles. Moreover, MPC exhibits minimal losses, enabling MPC-based lasers to achieve efficiencies of up to 96.4%^255^.

The MPC is well-suited for high-average-power laser pulses, offering high conversion efficiency, stability, and beam quality. The maximum pulse energy transmitted through a gas-filled MPC is only constrained by mirror fluence and gas ionization, scaling linearly with its length^265^. Besides exploring novel pulse parameter regimes, the MPC concept offers significant practical advantages. Essentially, it requires only two curved mirrors and a Kerr medium in between, making the method cost-efficient and easy to implement. Furthermore, MPCs are robust, largely insensitive to beam pointing, and capable of handling small mode mismatches without transmission losses^246^. Additionally, large B-integrals can be acquired without the need for extensive system lengths, rendering MPC a compact alternative to traditional broadening concepts. These advantageous properties have made MPC highly appealing, not only for scientific applications but also for commercial and facility laser systems where reliability is paramount^266^. Yet, compared to HCF and MPSC techniques, it remains a challenge to generate single-cycle pulses with MPCs. As the pulses pass through the multipass cell and their spectrum broadens, they become ever more sensitive to dispersion. Ensuring matching mirror dispersion for each pass will require delicate mirror design, adding to complexity and cost.

##### Cascading different spectral broadening techniques

The various spectral broadening methods discussed above can be combined to complement each other. For example, the high efficiency of aN MPC can be used to initially compress high power pulses down to few tens of femtosecond and, subsequently, using HCFs that offer simpler phase modulation across the ultrawide bandwidth, the pulses can be further compressed to few optical cycles^267,268^. To ensure optimal beam quality before compression, spatial filtering of the beam is first performed using an HPC, which stabilizes the beam waist position and size. This stabilization ensures periodic caustics throughout the entire channel of the MPC. The MPC subsequently completes the final pulse compression, producing ultrashort pulses with a duration of two optical cycles (~5.3 fs)^268^. Alternatively, by integrating the multiple thin plates approach directly inside an MPC, a compression factor of over 120 has been demonstrated^252^. Furthermore, the combination of MPC and MPSC techniques can also enable the generation of few-cycle pulses^269^.

#### OPA for few-cycle pulses

Optical parametric amplifiers have been introduced in the section “Amplification” for generating high-energy pulses. But they are also capable of generating pulses with few optical cycles^270^, significantly extending the wavelength tuning range^118^ and facilitating stable CEP control based on passive stabilization^271^. As discussed in the section “Amplification”, the crystal's damage threshold imposes a constraint on the increase of pulse peak power. To surmount this limitation, various techniques such as OPCPA, FOPA, and DC-OPA have been proposed, the principles of which are elaborated in section “Amplification”. These have led to outstanding experimental performance in generating few-cycle pulses: OPCPA^136,137,272^ and FOPA^158,273^ have produced laser pulses of less than two cycles, while DC-OPA has achieved nearly single-cycle laser pulses^150^. For a more detailed description, please see the section “Amplification”. Although QPCPA is theoretically capable of generating few-cycle pulses, no experimental verification has been reported to date.

#### Coherent pulse synthesis

Despite the great advances in post-compression techniques and optical parametric amplifiers in reaching the few-cycle regime, it remains a challenge to achieve single-cycle pulses based on these technologies, not least because of the difficulties in regulating the phase across such a wide spectrum. Coherent pulse synthesis technology represents a promising approach to overcome this limitation. It broadens the spectrum by coherently combining two or more parent pulses with different spectral components. By adjusting parameters such as relative phase, amplitude, and frequency of the input light waves, pulses that range from a few-cycle, single-cycle to even sub-cycle^43^ can be generated.

Coherent pulse synthesis is carried out via two primary approaches. The first involves synchronizing two or more independent lasers through active locking of their repetition frequencies and CEP offsets^274^. The second method involves synthesizing two or more pulses derived from the same pulsed source, each having undergone distinct nonlinear processes and consist of different spectral components^196,275–277^. In both cases, it necessitates not only femtosecond-level temporal synchronization of the pulse envelope, but also the stabilization of their underlying electric field—or equivalently, stabilizing the pulses' CEP. Coherent synthesis typically produces broadband pulses and can directly generate attosecond-scale pulses^44^. It can also be used to drive high harmonics^60^. However, coherent synthesis systems often require sophisticated optical and electronic components, which complicates system construction and maintenance. System stability may also be impacted by environmental factors such as temperature, humidity, and mechanical vibration. For a more detailed discussion of various coherent pulse synthesis techniques, readers are referred to ref.^278^.

#### CEP stabilization

**Fig. 12:**
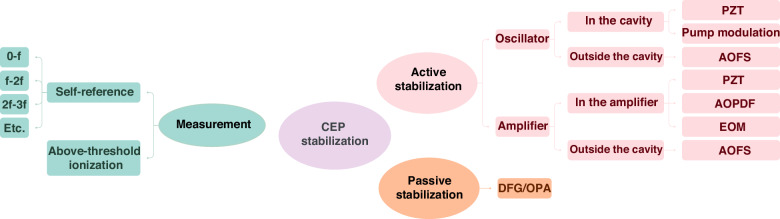
**Categorization of CEP stabilization schemes**

The realization of CEP-stabilized mode-locked lasers^279,280^, in particular via self-referencing techniques^281^, laid the foundation for the generation and measurement of reproducible attosecond pulses^1,2,18^. As the ultrafast driving laser's pulse duration is shortened to merely a few optical cycles, the CEP is highly influential and ensuring its stability is paramount. Measuring the absolute CEP of an individual pulse is a complicated task. On the other hand, the pulse-to-pulse variation in CEP can be inferred by measuring the carrier-envelope offset (CEO) which, while also not simple, is nevertheless more accessible. For a given CEO, or a given rate of slippage, the CEP will cycle back to the starting value after a specific number of pulses. By selecting only those pulses of the same CEP, as routinely done with pulse pickers in regenerative amplifiers, a train of CEP-stable pulses at a lower repetition rate can be obtained. Alternatively, if one can ensure the CEO is zero, then the entire train of pulses share the same CEP^280,282–286^. Characterizing and stabilizing the CEO thus represents an important first step of CEP stabilization. Figure 12 illustrates the main contents of this subsection. For more information, readers are referred to the extensive review by Cerulle et al.^271^.

##### Measurement

For mode-locked laser oscillators, the repeating pulses in the time domain form a series of equally spaced longitudinal modes in the frequency domain, where the frequency spacing equals the repetition frequency *f*_rep_, which is determined by the cavity length. A change in the CEP Δ*φ* in the time domain corresponds to a frequency shift in the frequency domain, defined as the CEO *f*_CEO_. Consequently, the frequency of the nth cavity mode is given by ${f}_{n}=n{f}_{rep}+{f}_{{\rm{CEO}}}=(n+\varDelta \varphi /2\pi ){f}_{rep}$^279^. In the special case where not only the *f*_CEO_ but also the *f*_rep_ is stabilized, one obtains a stabilized frequency comb.

The most commonly used methods for measuring the *f*_CEO_ involves self-referencing techniques that convert optical frequency signals, which are challenging to measure directly and accurately, into a radio frequency (RF) signal through interference. Among the most frequently adopted self-referencing methods are the *f–2f* interferometer method^57,218,280,287,288^ and the *0–f* interferometer method^289–291^.

**Fig. 13:**
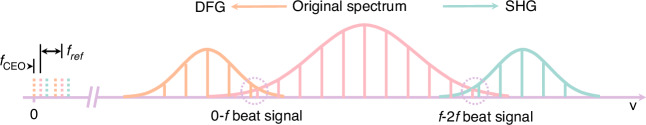
**Comparison between the 0–*f*** **and** ***f*–2*f*** **principles**^290^. Redrawn based on ref.^290^

The concept for the *f–2f* interferometer, also known as second-harmonic interferometry, is illustrated in Fig. 13. It extracts the CEO by frequency doubling the low-frequency components of a spectrum that covers at least one octave. These components are then coherently superimposed on the high-frequency components from the original spectrum. With the frequency of the low-frequency part denoted as${f}_{n}=n{f}_{rep}+{f}_{{\rm{CEO}}}$, its multiplied signal can be expressed as ${f}_{m}=2{f}_{n}=2(n{f}_{rep}+{f}_{{\rm{CEO}}})=2n{f}_{rep}+2{f}_{{\rm{CEO}}}$. Similarly, the frequency of the high-frequency part is denoted as ${f}_{2n}=2n{f}_{rep}+{f}_{{\rm{CEO}}}$, and the carrier-envelope offset *f*_CEO_ can be obtained by beating the frequency of $2{f}_{n}$ with ${f}_{2n}$, expressed in terms of the formula ${f}_{{\rm{CEO}}}=2{f}_{n}-{f}_{2n}=(2n{f}_{rep}+2{f}_{{\rm{CEO}}})-(2n{f}_{rep}+{f}_{{\rm{CEO}}})$. Although the *f*_CEO_ is often detected and locked to a non-zero value, it can also be stabilized to zero by a mixture of optical and electronic manipulation^280,282,286^.

The *0–f* interferometer method, also referred to as the self-differential frequency method, is employed for extracting the CEO information through difference-frequency generation (DFG). When considering frequency components as ${f}_{n}=n{f}_{rep}+{f}_{{\rm{CEO}}}$ and ${f}_{m}=m{f}_{rep}+{f}_{{\rm{CEO}}}$ (n > m) for self-differential frequency, ${f}_{{\rm{DFG}}}=(n{f}_{rep}+{f}_{{\rm{CEO}}})-(m{f}_{rep}+{f}_{{\rm{CEO}}})=(n-m){f}_{rep}$ is obtained and, if there is spectral overlap between the fundamental and the DFG spectra, the *f*_*DFG*_ is interfered with the pulse's own frequency components ${f}_{n-m}=(n-m){f}_{rep}+{f}_{{\rm{CEO}}}$. The beat signal obtained is the CEO ${f}_{{\rm{CEO}}}={f}_{n-m}-{f}_{{\rm{DFG}}}=(n-m){f}_{rep}+{f}_{{\rm{CEO}}}-(n-m){f}_{rep}$.

The *f*–2*f* method demands a pulse spectrum with a bandwidth of at least one octave. While such bandwidth can directly be generated from certain lasers, such as a Ti:Sapphire laser, it demands stringent control of the oscillator’s dispersion across a broad bandwidth, which is not a trivial task. Pulses with insufficient spectral width can benefit from similar methods such as the 2*f*–3*f*^292–294^ and higher-order alternatives such as the 7*f*–8*f* (or 3.5*f*–4*f*)^279^ method. For example, for 2*f*–3*f*, only 2/3 of an octave is needed. However, in higher-order schemes, both harmonics used for creating the beat-note are generated via nonlinear frequency conversion, that are less efficient for higher orders. This limits their optical power, making it more challenging for the resulting beat-note to achieve a signal-to-noise ratio comparable to that of lower-order setups^294^. Consequently, their applications are relatively limited.

In many cases, even with higher-order alternatives, external spectral broadening techniques are required prior to harmonic generation. This often includes coupling laser pulses into photonic crystal fibers (PCF)^295^ or other nonlinear waveguides to extend the spectrum beyond an optical octave. This usually entails temporal and spatial separation and recombination at interferometric precision for a strong interference signal on the detector. Consequently, it is sensitive to mechanical vibration and environmental noise, such as temperature and humidity drift.

The issues relating to the separation and recombination can be alleviated by obtaining the spectral broadening and the harmonic generation conversion within the same beam path^296,297^. Besides harmonic generation, difference-frequency generation can also be utilized for creating interference^289^ and in a monolithic approach^290,291^. This can also be understood as a 0–*f* interference, with the low-frequency component generated by SPM beats with the intra-pulse DFG components to generate a CEO signal. Figure 13 illustrates a comparison of the principles of the 0–*f* and *f*–2*f* methods. Yet, the 0–*f* method also faces various challenges. For the signal generated from intra-pulse DFG to be of high enough frequencies to overlap with the SPM-generated spectrum, there needs to be a large frequency difference between the high- and low-frequency edges of the input spectrum. Furthermore, to ensure the high- and low-frequency components for DFG arrive simultaneously at the crystal for interactions, careful management of dispersion, preferably in the form of a well-compressed pulse (<5 fs), would be required. In general, monolithic schemes offer reduced possibilities for optimizing the intensity of the beat frequency signal. Thus, mainstream commercially available CEO-locked oscillators continue to adopt the *f*–2*f* method.

The generation of the *f*–2*f* interference beat-note in the radiofrequency domain requires a train of pulses incident on the photodetector. This works well for the high (>MHz) repetition rate of a laser oscillator. But for the typically low (<kHz) repetition frequency of laser amplifiers, it is challenging to obtain the needed beat-note strength within a reasonable time before the CEP has already drifted. Therefore, a different CEP measurement technique is required, namely by analyzing the spectral fringes in the optical spectral domain where the *f*–2*f* components spectrally and spatially overlap, but are separated by a temporal delay^57,287,298^. This allows single-shot measurement of the CEP phase and is typically employed to track changes in CEP between pulses when the fluctuations in their relative phase and group delay are not excessive.

Apart from spectral interference, various other techniques have been explored and developed. These include linear optical interferometry^299^ and quantum interference in photocurrents^300,301^, which can determine the variation in CEP. The absolute CEP can be determined with phase-sensitive measurements, such as photo-emission^302–304^ and photoionization^216,223,305–307^, the latter can provide single-shot measurements^308–310^. Attosecond streaking^311^ and other field sampling techniques^312–319^ also provide direct access to the CEP. In particular, in wide bandgap solids, the excitation of electrons into the conduction band and their light-field-induced acceleration can produce an electric current, whose direction and amplitude associate with the CEP of the driving laser field. Such setups can operate under ambient condition without complex vacuum setups, and the required pulse energy is at µJ-level^312,313^ and as low as nJ for layered materials^319^. Just as excitingly, phase-sensitive techniques are also being rapidly developed with chip-scale nano-structures^320–327^, further reducing the required pulse energy to pJ-level.

##### Active CEP stabilization

The active stabilization of CEP involves measuring the CEO offset of the laser pulse and manipulating either the laser cavity emitting the pulse or directly the laser pulse itself. The methods for oscillator and amplifier CEP stabilization often differ. For oscillators, a “fast-loop” with high feedback bandwidth (>10 s kHz) is typically used. In 2000, David J. Jones and Scott A. Diddams, et al., implemented a method where the offset signal was fed back to a cavity mirror affixed to a piezoelectric ceramic (PZT)^280^. The control signal stabilizes the CEP by tilting the end mirrors or adjusting the insertion of wedges in the cavity^279,280,328–330^. Tilting the end mirror, given the spatial dispersion deliberately introduced in the cavity design, induces a linear phase change with frequency, modifying the dispersion and the corresponding group delay of the pulse^279,280^. Adjusting the wedges can also regulate the delay between group and phase velocities, thereby controlling the CEP. However, the feedback bandwidth of these methods is typically in the tens of kHz, and only recently reaching hundreds of kHz^331,332^.

The second control approach and currently the prevailing method involves stabilizing the CEP through pump power modulation using either an electro-optic modulator (EOM) or acousto-optic modulator (AOM)^57,333^ or, especially when the laser is directly pumped using laser diodes, by modulating the diode current^334–336^. Modulating the pump power or, more generally, the gain dynamics^290,333,337–339^ can influence the nonlinear phase shift caused by self-phase-modulation in the gain medium, resulting in disparate phase and group velocity shifts^330,340^. The feedback bandwidth of this method is commonly at hundreds of kHz, surpassing that of a PZT and is primarily constrained by the upper state lifetime of the gain crystal and thereby the response time of the laser. With such methods, residual CEP noise can reach <7 mrad^341,342^. Other methods of controlling the intracavity power include loss modulation^343–345^, which can extend the locking bandwidth above the laser lifetime limit.

**Fig. 14:**
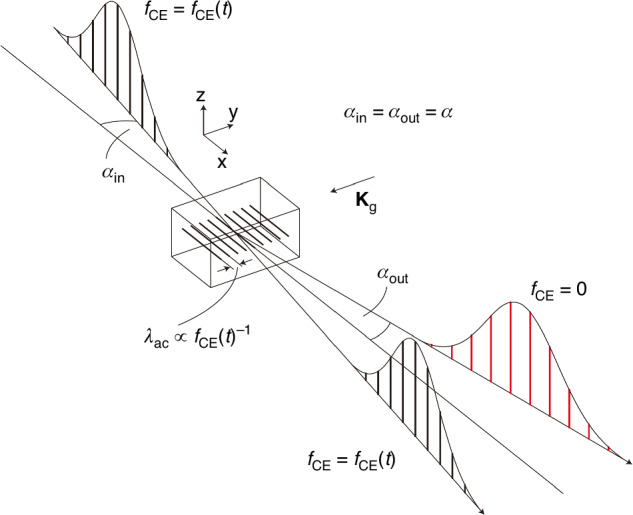
**Concept of the direct feed-forward method for stabilization of CEP**^284^. *f*_CE_ is the carrier-envelope offset frequency. Reproduced from ref.^284^, with permission from Springer Nature. Copyright © 2010 Springer Nature

**Fig. 15:**
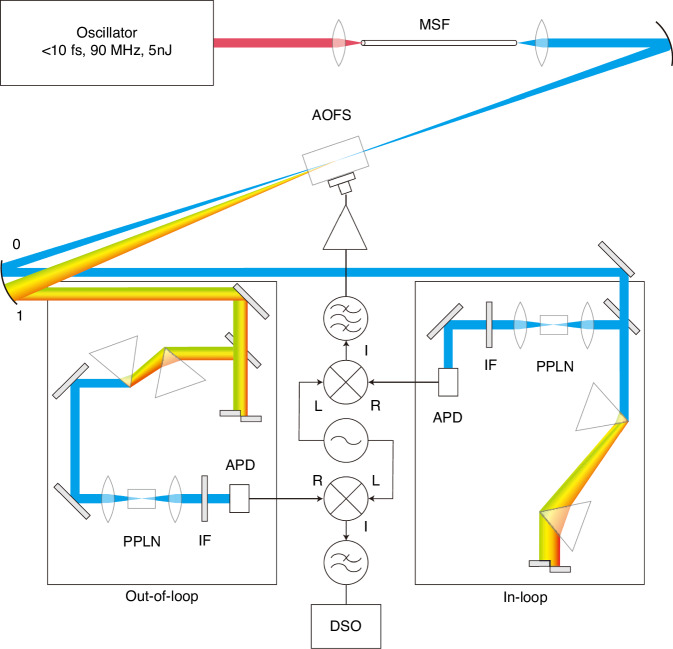
**An experimental setup used to stabilize CEP based on AOFS**^284^. MSF microstructured fiber, AOFS acousto-optic frequency shifter with numbered output orders, PPLN periodically poled lithium niobate crystal, IF interference filter, APD avalanche photodiode. DSO digital sampling oscilloscope. Interferometers use a quasi-common path geometry with a split end mirror for compensation of the group velocity dispersion in the MSF and subsequent optical components. The setup shown incorporates an additional frequency synthesis step using an external frequency generator, two double-balanced mixers (R, RF input; L, local oscillator input; I, intermediate frequency output) and suitable RF filters. This step is only required for unambiguous and sensitive phase noise analysis. Reproduced from ref.^284^, with permission from Springer Nature. Copyright © 2010 Springer Nature

Another scheme for stabilizing the oscillator CEP involves a feed-forward method using an acousto-optic frequency shifter (AOFS)^284^. The feed-forward method modulates the phase outside the cavity without impacting the oscillator itself. This principle is illustrated in Fig. 14. Diffraction off the index grating inside the AOFS splits the frequency comb of a free-running femtosecond laser oscillator into the zeroth-order and first-order beams. While the comb mode in the transmitted beam (zeroth order) remains unaffected, each individual frequency of the diffracted beam undergoes a shift by the driver frequency *f*_RF_ of the AOFS. If *f*_RF_ equals *f*_CEO_ plus an integer multiple of the laser repetition frequency, the comb can be shifted to the zero-offset frequency. Figure 15 depicts the experimental setup for CEP stabilization based on this principle. The stabilization device primarily comprises of an in-loop setup, with an optional out-of-loop for independent stability characterization. The in-loop acquires the offset signal *f*_CEO_ from the zeroth order or the unmodified oscillator output beam, and feeds the signal directly into the AOFS, either without further frequency synthesis, or by adding an integer multiple of the laser repetition frequency. The out-of-loop retrieves the offset signal by detecting the first order, allowing for independent optical and electronic analysis of the residual phase noise^284^.

In contrast to the conventional electronic phase-locked loop feedback technique, the feed-forward method can operate without a PID loop^346^, offering a broader bandwidth and can more effectively compensate high-frequency phase noise. Furthermore, the control loop remains external to the oscillator cavity and does not alter the parameters within. This technology can achieve very low integrated phase noise of tens of mrad^284,346^ and even 3.5 mrad when applied to a low-timing-jitter laser^347^. Combined with techniques for compensating for long-term drifts, the lock can be sustained for over 75 h^348^. Nevertheless, the AOFS introduces material dispersion and, due to diffraction, relatively high losses and angular dispersion. These make the technique less attractive for ultra-broadband few-cycle pulses.

Despite progress in stabilizing the oscillator's CEP, the amplified CEP remains susceptible to slow drift due to factors such as beam pointing instability, temperature fluctuations, environmental vibrations, and variations in pump light energy, necessitating additional locking of the amplifier's CEP. The amplifier's typically much lower repetition frequency means the stabilization of its CEP is often designated as the 'slow loop'.

The methods for actively stabilizing the CEP of amplifiers can be categorized into three main types. One approach involves adding the feedback signal from the amplifier to the oscillator's CEP stabilization scheme and preemptively compensating for phase distortions arising in the amplifier^57,280,336,349^. However, the coupling between the fast and slow loop signals in the oscillator can result in a less stable lock.

The second method involves adjusting the internal device of the amplifier to independently lock the CEP of both the oscillator and the amplifier. Three common devices used to stabilize the amplifier's CEP include PZT, acousto-optic programmable dispersive filter (AOPDF), and EOM. The PZT enables fine tuning of the grating spacing to compensate for dispersion, thereby achieving CEP stabilization of the amplifier^350–353^. The method utilizing the AOPDF, proposed as early as 1997^354^, sends a feedback signal into the AOPDF to provide dispersion compensation and modulate the phase for CEP stabilization^355,356^. The AOPDF offers a wide dispersion compensation range and is compact in size, requiring only minor modifications for implementation in existing CPA systems^357^. The third device, the EOM, applies a moderate voltage to a crystal with non-zero electro-optics response (such as LiNbO_3_) and induces a corresponding change in refractive index^358^. This also changes the difference between the phase and group velocities, thereby achieving CEP stabilization^264,288,359,360^. This method is relatively simple, permits large phase shifts, and increasing the number of crystals enhances the amplitude of CEP correction. Additionally, its response time is very short, typically ranging from nanoseconds to picoseconds (for fiber-based options), enabling CEP stabilization of high repetition rate amplifiers. The final method is to control the CEP after the amplifier through AOFS. It shares the same principles and benefits as outside-the-cavity control for oscillators^361^.

##### Passive CEP stabilization

The passive stabilization technique for CEP is an all-optical approach that does not need an external feedback loop. Instead, it leverages the phase relationship between individual optical components within a nonlinear optical process^362^. In DFG and OPA processes, the relationship between the signal, idler and the pump's phases, represented by $\varDelta {\varphi }_{signal}$, $\varDelta {\varphi }_{idler}$ and $\varDelta {\varphi }_{pump}$, is $\varDelta {\varphi }_{idler}=\varDelta {\varphi }_{pump}-\varDelta {\varphi }_{signal}-\frac{\pi }{2}$. When the signal and pump light share the same shot-to-shot CEP $\varDelta \varphi$, $\varDelta {\varphi }_{pump}=\varDelta \varphi +{c}_{1}$, $\varDelta {\varphi }_{signal}=\varDelta \varphi +{c}_{2}$, where *c*_1_ and *c*_2_ are constant phase offsets, the CEP $\varDelta {\varphi }_{idler}=const$ ^271,278,363^.

Passive CEP stabilization, in contrast to active methods, is an all-optical technique that operates without electronic feedback circuits. It directly produces a train of pulses with identical CEP, eliminating the need for pulse selection^271^. Furthermore, due to the characteristics of nonlinear optical processes, passive CEP stabilization offers a broader range of achievable central wavelengths and enhances system stability. However, in DFG schemes where the pump and signal have different beam paths, a slow stabilization loop still needs to be implemented to correct for drifts in path lengths. For DFG schemes based on intra-pulse interactions^271^, the CEP is fixed, and additional optical elements are usually needed to vary it for experiments. A recently reported scheme allows for changing of the CEP by taking advantage of the different CEP dependence in cascaded intra-pulse DFG^290^. Yet this requires a tunable CEP frontend in the first place.

### Longer-wavelength lasers—for water window attosecond pulses

The cut-off photon energy of the high harmonics generated from a gas medium can be calculated as ${(\hslash \omega )}_{\max }\approx {I}_{p}+3.17{U}_{p}$, where *I*_p_ is the atomic ionization potential, and ${U}_{p}={e}^{2}{E}^{2}/4m{{\omega }_{0}}^{2}\propto {\lambda }^{2}$ s the mean kinetic (or ponderomotive) energy of the electron quivering in the laser field, *E* is the amplitude of the electric field, *ω*_*0*_ is the laser frequency, and *e* and *m* are the charge and rest mass of the electron, respectively^52,78^. Consequently, the cut-off photon energy is proportional to the square of the wavelength of the driving laser. This can also be understood in a simplified picture. Since the optical cycle lasts longer for longer-wavelength driving lasers, the freed electrons after tunnel ionization have more time to accelerate and acquire higher kinetic energies from the driving laser field before recombining with the parent ions. This results in higher cut-off photon energies^39,364^.

The generation of ultrashort driving pulses and the resulting IAPs has initially been dominated by Ti:Sapphire laser technologies due to their broad gain bandwidth^59,60,365^. However, the emission wavelength for Ti:sapphire lasers lie in the near-infrared, limiting the photon energy of the attosecond pulses to typically less than 100 eV^365^. Soft X-rays in the range between 282–533 eV (2.3–4.4 nm) are absorbed by carbon and nitrogen—key components for organic chemistry—but practically not by water, which is often also present in biological samples at high concentrations. Thus, light sources for this “water window” is of great interest for studying the chemical constituents of life.

Soft X-ray pulses covering the water window can be generated utilizing a few-cycle mid-infrared (MIR) laser with stable CEP as the driving laser. Therefore, significant efforts have been devoted to the development of long-wavelength driving lasers. For instance, 1.8 µm^366^, 1.85 µm^367^, and 3.9 µm^95^ have been used to generate X-ray supercontinua that spans the entire water window and beyond.

Besides spectral coverage, the generated pulses also exhibit attosecond pulse durations, allowing dynamics of matter to be studied at attosecond time scale. Using longer-wavelength drivers is also beneficial for reducing the inherent group delay dispersion (attochirp)^368–370^. As an electron's trajectory in the continuum—and thus its emission frequency at recombination—depends on the time of its release relative to the optical cycle, different harmonic components making up the same attosecond pulse are generated at a slightly different time, leading to an intrinsic spectral phase difference. The propagation time of the electron in the continuum, and thus the emission time of successive harmonics, is proportional to the fundamental laser period *T*, while the harmonic energy is proportional to *U*_*p*_. Thus, the absolute value of the chirp (the second-order spectral phase) scales as $\beta \propto T/{U}_{p}$ and is inversely proportional to λ^369^. This fact suggests that driving lasers with longer wavelengths produces smaller attochirp and can produce shorter attosecond pulses.

Research on IAPs evolved significantly around 2010, when Ti:sapphire lasers were overtaken by driving lasers with longer wavelengths and MIR OPA entered the mainstream^39^. OPAs are ripe with possibilities for generating femtosecond pulses tunable over a wide range spanning 0.2–20 μm^121–123,371–376^. Optimized OPCPA^377,378^, DCOPA^379^, FOPA^273^ based on OPA are also popular choices for generating long-wavelength lasers. Based on different crystals, OPAs and their cascaded variants can generate high-power pulses at different wavelengths, with spectral broadening and pulse compression if few-cycle pulses are needed.

#### OPA and their cascaded variants

In the 1–5 μm range, the boron–oxygen compound BiB_3_O_6_, ((BIBO)^377,380^, LiB_3_O_5_ (LBO)^381,382^, *β*-BaB_2_O_4_ (BBO)^383,384^, potassium titanium arsenate oxide (KTiOAsO_4_, KTA)^208,385–387^, periodically poled lithium niobate (PPLN)^136,388^, MgO-doped congruent LiNbO_3_ crystals (MgO:LiNbO_3_, MgO:LN)^379^ are excellent nonlinear crystals for building OPAs^377,380^. Not only do they have large effective nonlinear coefficients, high damage thresholds and are not susceptible to deliquescence, they also have a very wide phase-matching bandwidth. Using an OPCPA based on such crystals and pumping at near 1.7 µm^137^, soft X-rays crossing into the water window has been demonstrated with a pulse duration of 53 as^389^. Using a driving laser at a wavelength of 3.9 μm, a HHG supercontinuum spanning from the ultraviolet to over 1.6 keV has been demonstrated. This bandwidth could support pulses as short as 2.5 attoseconds^95^. However, due to the limited transmission range of these crystals, it was not possible to employ driver pulses at longer wavelengths.

Above 5 μm, oxide nonlinear crystals are generally no longer applicable. ZnGeP_2_ (ZGP) crystal has high nonlinear coefficients, high damage thresholds, high thermal conductivity, and transmittance windows ranging from 0.64 to 12 μm^390^, making it very promising for generating few-cycle pulses in the mid-infrared region^391–394^. However, ZGP's transmission cutoff requires a pump wavelength >1.9 μm^395^, ruling out mature pump lasers near l μm such as Ti-, Yb- and Nd- laser systems. It necessitates the development of powerful 2-μm pump sources^396^ such as picosecond Ho-doped laser systems^397–399^. In contrast, lithium thiogallate (LiGaS₂)^400^ not only possesses a high damage threshold and wide transmission window but also effectively avoids two-photon absorption in lasers near 1 µm, enabling the use of well-established 1 µm pumps^401^. One drawback is its significantly lower nonlinearity and thereby conversion efficiency compared to ZGP.

#### DFG and intra-pulse DFG (IPDFG)

In addition to pumping an OPA with longer-wavelength pulses, DFG and IPDFG, are important techniques for producing long-wavelength light. DFG generates broadband mid-infrared pulses spanning several microns by mixing two tunable-wavelength lasers within a nonlinear crystal. IPDFG, a specialized form of DFG, mixes the different frequency components within an ultrabroadband few-cycle pulse to emit mid-infrared radiation. The IPDFG approach offers enhanced system stability and inherent CEP stabilization in a compact form.

Advances in OPCPA technology have enabled DFG systems to generate intense mid-infrared laser pulses^402–409^ with durations spanning only a few optical cycles^402,406,408,409^ and energies reaching hundreds of microjoules^404^. The generation of mid-IR pulses via IPDFG^410–413^ generally utilizes broadband ultrashort pulses as the pump source, with pump wavelengths ranging from the near- to mid-infrared (e.g., 1 μm^414^, 2 μm^410^, and 3 μm^415^). Although the output energy is typically limited to the low microjoule level, IPDFG offers a simpler setup capable of producing highly stable, intrinsically CEP-stable mid-infrared pulses. Furthermore, both DFG and IPDFG allow adjustments of the generated mid-infrared pulse envelope or the underlying waveform^297,416–419^, and their outputs could be further amplified^407^. Continuous improvements in pump sources, nonlinear crystals and the development of hybrid architectures are expected to enhance the roles of DFG and IPDFG in high-energy mid-infrared light sources.

#### Direct lasing and amplification with transition-metal-doped II-VI chalcogenides

In addition to nonlinear frequency conversion, direct lasing and amplification is another approach to generate high-power, few-cycle driving lasers in the 2–10 μm. Compared to OPA systems that consist of multiple and cascading stages, this method can be more efficient, simpler to operate and potentially less costly.

Cr- and Fe-doped chalcogenides^394^, represented by Cr:ZnS (Se) and Fe:ZnS (Se), are promising materials for direct lasing in the mid-infrared region^104,394^. They have already seen their first utilization in mJ-level femtosecond lasers in the 2–3 μm^420–422^ and 3–7 μm^423^ spectral ranges, employed as amplifier crystals seeded by nonlinearly frequency down-converted light from other lasers. An ultrafast master-oscillator power-amplifier system in which the oscillator and amplifier share the same gain material, such as Cr:ZnSe^424,425^, further simplifies the laser architecture.

Both Cr- and Fe-doped chalcogenides are vibronic laser crystals and share many characteristics with Ti:sapphire crystals, such as broad absorption and emission bandwidths^104^. The absorption band of Cr:ZnS(Se), in particular, overlaps with various readily available high-power pump sources, ranging from erbium- or thulium- doped fiber lasers to laser diodes^426–429^. This aided the rapid development and applications of Cr-doped chalcogenide lasers.

Since the first demonstration of femtosecond Cr:ZnS(Se) oscillator^430^, its performance has been steadily improved. The average power directly emitted from an oscillator can approach 2 W^431,432^, while peak power is approaching 1 MW^432,433^. The directly emitted pulse duration has also been shortened to below three optical cycles^297,342,434,435^. By employing low-noise pump sources, the intensity noise is sufficiently low^342,436^ to enable the stabilization of the CEO^436^. Incorporating a newer offset-free self-referencing technique^286^, CEP stabilization (CEO = 0) has also recently been demonstrated^297^, offering CEP phase noise that is the lowest for few-cycle laser sources regardless of wavelengths^342^. In addition, the offset-free technique allows the absolute CEP to be varied without adjusting optical elements downstream^297^, thereby avoiding the introduction of unwanted dispersion and waveform distortion in phase-sensitive experiments. The oscillator repetition rates—important for frequency comb applications—have also reached above GHz-level^437,438^.

Apart from oscillator development, amplification at MHz repetition rates has been showcased at multi-watt-level^433,439^ and at low noise^429^, proving the ability for the gain medium to handle high average power while maintaining the CEO stability^436^. Together, these different developments consolidate the foundation for building the next-generation, highly stable yet manipulatable long-wavelength HHG drivers using Cr-doped ZnS (Se) media.

With Fe:ZnS(Se), progress has also been made as high-power pump source near 3µm, such as Er:YAG^440^, Er:Y_2_O_3_^441^, Cr:ZnSe^442,443^, and Er:ZBLAN^444^ lasers, become available. This led to the first demonstration of femtosecond pulses from a mode-locked Fe:ZnSe oscillator operating at 4.4 µm in 2020^445^. As the underlying technologies mature for both Cr- and Fe-doped chalcogenides—often described as the Ti:Sapphire of the mid-infrared—they are likely to form an important pillar in future attosecond technologies.

### High-repetition-rate lasers for high-repetition-rate attosecond pulses

#### Enhancement cavities

The low conversion efficiencies for HHG, especially when driven at longer wavelengths, mean the photon flux per attosecond pulse is severely constrained. Moreover, currently available driving lasers for attosecond sources typically operate at a repetition rate limited to the kHz regime^51,98,446,447^. Taken together, the low overall photon flux significantly limits signal-to-noise ratios and leads to impractically long measurement times. The use of machine learning promises to help improve noise tolerance^448^ and the recovery of spectral information^449^. In particular, multimodal data synergy can enhance analytical precision^449,450^. Yet their development is still in the early phase. Furthermore, many experiments require low-flux attosecond pulses to preserve measurement accuracies. For example, to avoid space-charge effects when measuring photoelectrons and ions originating from attosecond dynamics, the flux per attosecond pulse needs to be restricted^35,451,452^.

**Fig. 16:**
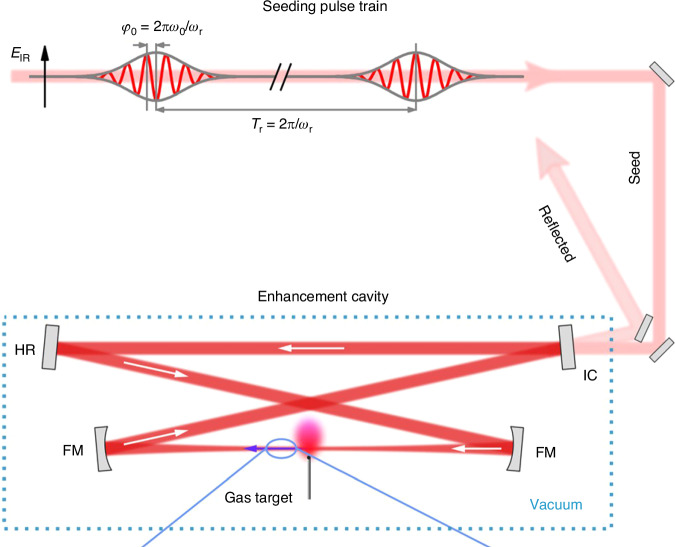
**Schematic of a cavity-enhanced HHG setup**^454^. Adapted from ref.^454^, with permission from Springer Nature. Copyright © 2021 Springer Nature

Consequently, the development of high-repetition-rate attosecond lasers will spur significant advancement in attosecond physics. Presently, there are two primary methods for attaining high repetition frequency attosecond pulses^453^. A promising approach involves HHG within a resonant enhancement cavity. In these setups, driving pulses with low energies (~μJ) but at very high repetition rates (>10 MHz) are employed. The operational mechanism of cavity-enhanced HHG is illustrated in Fig. 16^454^. A phase-stabilized laser system emits visible/near-infrared pulses at multi-megahertz repetition rate, which are subsequently coupled into a free-space, passive optical resonator using a partially transmitting input coupling mirror. Typically, the resonator’s stability criteria are designed to minimize diffraction losses for defined transverse eigenmodes. To ensure unidirectional output for the high harmonics generated, a ring resonator configuration is commonly utilized. Constructive interference between the input pulse's electric field and that of the circulating pulse at the input coupler is facilitated by active locking, thus affording effective coupling of the seeding pulse train into the passive resonator. When resonance conditions are met and the round-trip loss in the resonator is sufficiently low, the energy of the circulating pulse can surpass that of individual input pulses by several orders of magnitude. This energy amplification enables efficient HHG at repetition rates reaching tens of MHz.

The first demonstrations of cavity-enhanced HHG boosted the peak-power of femtosecond pulses from Ti:sapphire oscillators to >3 × 10^13^ Wcm^−2^ and produced frequency combs in the XUV (seventh harmonic)^455^ and the EUV (15th harmonic)^456^. The repetition frequencies of over 100 MHz represent three orders of magnitude improvements over previous reports. The frequency comb structure with large frequency mode spacing open the way for high resolution spectroscopy in these important wavelength regions^457–459^ (see also the review paper by Pupeza et al.^454^). The technique has recently led to the precise measurement of the long sought-after transition frequency of ^229m^Th, which opens the way for the building of a nuclear clock^460^. As the field progressed, Ti:sapphire lasers gradually gave way to high-repetition-rate, high-average-power Yb:fiber CPA lasers^461^. Pulse repetition rates in these systems range from 10 to 250 MHz^462,463^, with lower rates limited by resonator length and higher rates facing efficiency challenges from cumulative plasma effects^464,465^.

However, the generation of harmonics directly within the resonator necessitates an output coupling mechanism, adding additional complexity. Furthermore, the ionization of gases perturbs the circulating pulses and imposes additional constraints on the phase-matching conditions and attainable enhancements^461,466–468^. The generation of IAPs has so far never been experimentally demonstrated using enhancement cavities. However, in 2017, M. Högner et al. theoretically predicted the feasibility of efficiently generating IAPs at multi-MHz repetition rates via cavity-enhanced HHG^469^, followed by experimental demonstrations of the underlying mechanisms^470,471^. They predicted IAPs with photon energies at approximately 100 eV and a photon flux of at least 10^8^ photons per second at repetition rates of 10 MHz and higher, employing 0.7 mJ, five-cycle pulses from the seeding laser coupled with a state-of-the-art enhancement cavity. Additionally, they introduced a novel method, termed transverse mode gating (TMG), which utilized non-collinear optical gating within a tailored transverse cavity mode.

#### High-repetition-rate, high-power lasers

Another approach for generating attosecond pulses at higher repetition rate (100 kHz) focuses on improving single-pass HHG^453^. For conventional gas-based high harmonics, great efforts have been made to raise the pulse energy of the high repetition rate driving laser to a level comparable to earlier kHz systems (~mJ), allowing for the retention of the pump-probe capability while increasing the repetition rate.

Ti:Sapphire amplifier systems have traditionally generated short, high-energy pulses at repetition frequencies ranging from 10 Hz to 10 kHz, constrained primarily by thermal effects^94,472–474^. The repetition frequency of the IAPs generated by the Ti:Sapphire CPA laser was also limited to less than 10 kHz^365,475,476^ due to excessive complexity and cost, inevitably compromising long-term stability and robustness.

In comparison, OPCPA has the capability to generate CEP-stable, few-cycle pulses with multi-μJ-level energy, at repetition rates reaching hundreds of kHz^451,477,478^. Recently, based on an OPCPA laser system, Witting et al. demonstrated for the first time IAPs at 100 kHz with a duration of less than 140 as for high numbers (10^6^) of XUV photons per pulse^479^. The direct nonlinear post-compression of laser pulses from high-power, high-repetition rate fiber chirped pulse amplifier has also emerged as a valuable alternative to OPCPA to increase the photon flux. Through fiber CPA with post-compression, the repetition frequency of the laser pulse can be escalated to megahertz level, and the higher harmonics photon flux can reach 10^13^ photons per second^466^. Additionally, achievable APT's repeat frequency can be boosted to 100 kHz based on fiber CPA with post-compression^453,480^.

Single-pass HHG driving lasers are mainly reliant on amplifier systems, which increases complexity and requires a larger footprint. Ultrafast ytterbium-doped thin-disk laser oscillators present a feasible alternative, offering adequate average and peak power for HHG within its compact cavity^481–484^. They can achieve repetition rates in the megahertz range^485–488^, enabling the generation of intracavity high harmonics of single-pulse photons exceeding 10^8^ photons per second^489^. Although thin-disk laser oscillators have not yet been used to produce attosecond pulses experimentally, their low noise performance and potential power scaling make them a promising candidate to become the next-generation attosecond driving laser, potentially delivering significantly shorter acquisition times and improved signal-to-noise ratios.

For solid-based high harmonics, the required focusing intensity is much lower than that for HHG in gases. This allows for the generation of high harmonics using driving lasers with high repetition rates on the order of low-energy nanojoules^490–492^. With continued technological advancements, this approach may also lead to the generation of high-repetition-frequency attosecond pulses.

## Summary and outlook

The arrival of the attosecond era has spawned countless exciting applications. Satisfying the diverse demands requires bespoke attosecond pulses. We reviewed the principles and historical development of various driving lasers for attosecond generation according to four key aspects: pulse energy, pulse width, wavelength, and repetition rate. First, we recounted the primary methods for obtaining high pulse energies: direct or parametric amplification and coherent pulse synthesis. Secondly, we described methods for generating few-cycle pulses, including nonlinear post-compression techniques and OPA variants. We also discussed the various schemes for CEP stabilization to generate IAPs. Thirdly, we introduced lasers capable of emitting at different wavelengths, highlighting the trend towards the mid- to far-infrared regions for generating attosecond pulses with higher photon energies. Finally, we introduced laser architectures capable of supporting high-repetition-frequency, few-cycle output.

With the continued advancement of attosecond science in frontier fields such as strong-field physics, ultrafast spectroscopy, and high-resolution imaging, driving lasers are rapidly evolving to meet increasingly stringent requirements on attosecond pulse characteristics in the temporal, spectral, and energy domains. In the coming years, next-generation driving laser systems are expected to achieve major breakthroughs in the following areas: First, increasing the single-pulse energy of table-top systems to the multi-millijoule level and beyond will expedite the exploration of strong nonlinear interactions in high-density plasmas and novel media under extreme-field conditions. Second, the development of long-wavelength driving lasers will allow further extension of the HHG cutoff into the soft X-ray regime, not only enabling studies in the water window, but also benefiting techniques such as high harmonic spectroscopy for molecular orbital imaging. Third, the realization of high-repetition-rate (100 kHz–MHz) laser systems will be crucial for meeting the demanding requirements of attosecond pump–probe experiments and multidimensional correlation measurements, particularly in terms of average power, sampling efficiency, and signal-to-noise ratio. Moreover, in terms of system architecture, by integrating hybrid OPA designs with, for example, thin-disk amplifier platforms that offer efficient thermal management, future driving sources are expected to become more compact and robust.

Despite substantial advancements in related technologies, several critical bottlenecks persist. Chief among them is thermal load management in high-average-power systems, which remains essential for ensuring long-term operational stability. Secondly, achieving robust CEP stabilization at pulse energies exceeding 10 mJ, particularly in long-wavelength platforms or multi-stage amplification architectures, continues to be a major challenge. Furthermore, the limited availability of broadband, high-damage-threshold mid-infrared optical components, such as nonlinear crystals and anti-reflection coatings, poses a significant barrier to further performance enhancement. Addressing these technological challenges will not only enable the refinement and wider deployment of attosecond light sources but also pave the way toward the exploration and realization of zeptosecond lasers, pushing the frontiers of ultrafast science in both spatial and temporal domains.

## Data Availability

All data were available from the corresponding authors upon reasonable request.
